# Supplementary material for: UBR5 promotes antiviral immunity by disengaging the transcriptional brake on RIG-I like receptors
Source: Nat Commun. 2024 Jan 26;15:780. doi: 10.1038/s41467-024-45141-1 (PMC10817939; doi:10.1038/s41467-024-45141-1)

UBR5 promotes antiviral immunity by disengaging the transcriptional brake on RIG-I like receptors

Duomeng Yang, Tingting Geng, Andrew G. Harrison, Jason G Cahoon, Jian Xing, Baihai Jiao, Mark Wang, Chao Cheng, Robert E. Hill, Huadong Wang, Anthony T. Vella, Gong Cheng, Yanlin Wang, Penghua Wang

This Extended Data contains 11 figures and legends, and uncropped blots.

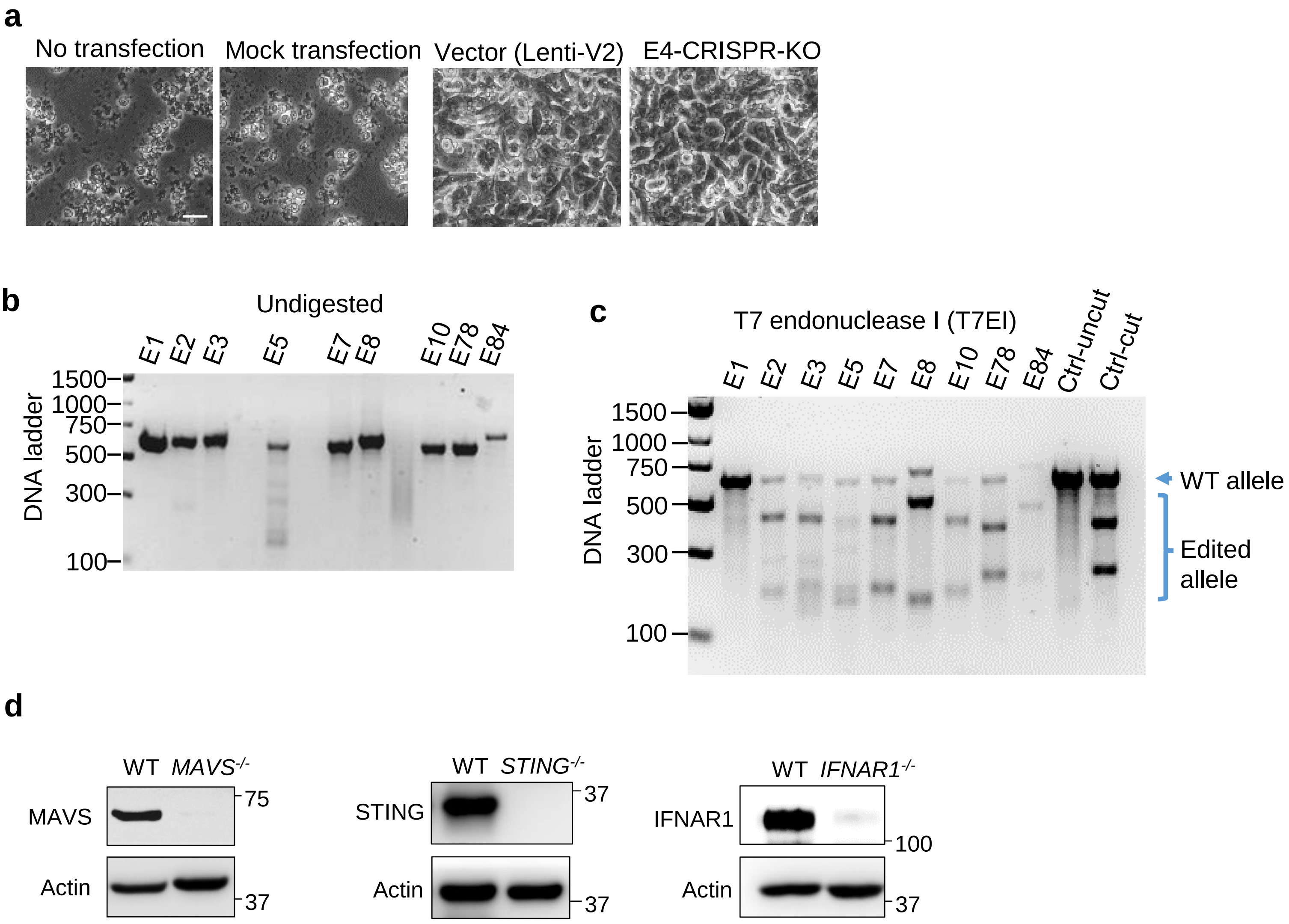

Extended Data Fig. 1. Generation of E3 ligase knockouts by CRISPR-Cas9 in 2fTGH-ISRE-Luc cells. Related to Fig.1. a) Selection of lentivirus-transduced stable cells with 1 µg/mL of puromycin for 6 days. The non-transduced cells (No and Mock transfection) were killed completely by day 6 after puromycin was added, while the transduced cells (Vector and E4) survived and proliferated, scale bar: 50 µm. **b, c)** T7 endonuclease I (T7EI) mismatch cleavage-assay. A genome fragment (~ 600 bp) was amplified by PCR with gene specific primers flanking the guide RNA-targeted site. **b)** shows the undigested PCR products. **c)** shows the digested PCR products by T7EI. Control (ctrl) is a positive in the assay kit. **d)** The immunoblots show MAVS/STING/IFNAR1 knockout in 2fTGH-ISRE-Luc cells.

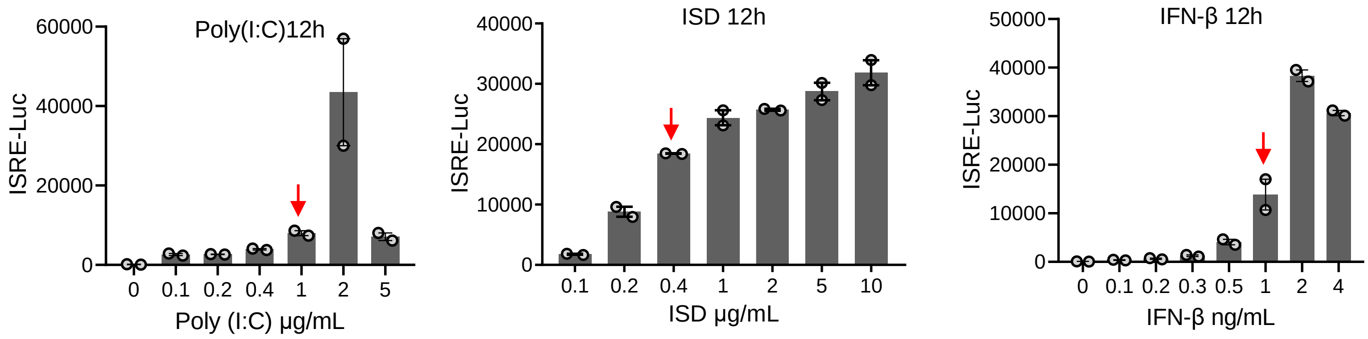

Extended Data Fig. 2. Optimization of the ligand doses. Related to Fig.1. 2fTGH-ISRE-Luc cells (~70% confluency) were transfected with different concentrations of poly (I:C) or ISD, or treated with recombinant human IFN-β for 12 hr. The ISRE activity was quantitated with a luciferase reporter assay. The arrows indicate the optimal concentration. Data are presented as mean ± S.E.M, *n*=2 biologically independent experiments. Source data are provided as a Source Data file.

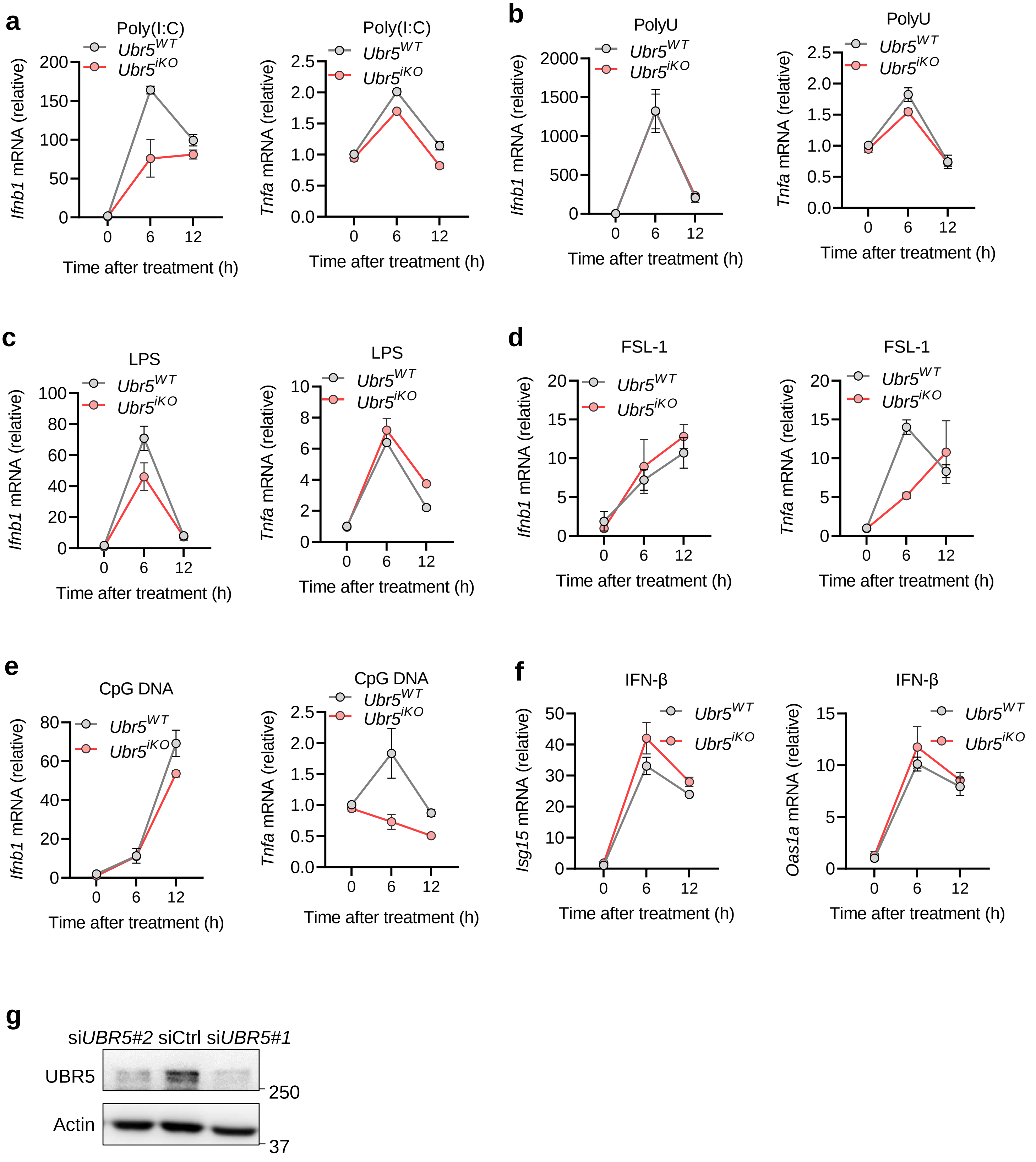

Extended Data Fig. 3. UBR5 is dispensable for TLR and JAK-STAT1/2 signaling. **Related to Fig.2.** Bone marrow derived macrophages were treated with **a)** poly(I:C) (no transfection, to stimulate TLR3), **b)** transfected with polyU (TLR7), **c)** treated with LPS (TLR4), **d)** FSL-1 (TLR2/6), **e)** transfected with CpG DNA (TLR9), or **f)** treated with recombinant mouse IFN-β (JAK-STAT1/2). The indicated gene transcripts were quantified by qRT-PCR and normalized with the house keeping beta actin. **g)** The immunoblots show UBR5 depletion by siRNA (siCtrl) or UBR5 siRNA (*siUBR5#1* and *#2*) for 48 hr. Data shown in **a**-**f** are presented as mean ± S.E.M, two-tailed student's *t*-test, *n*=2 biologically independent experiments. Source data are provided as a Source Data file.

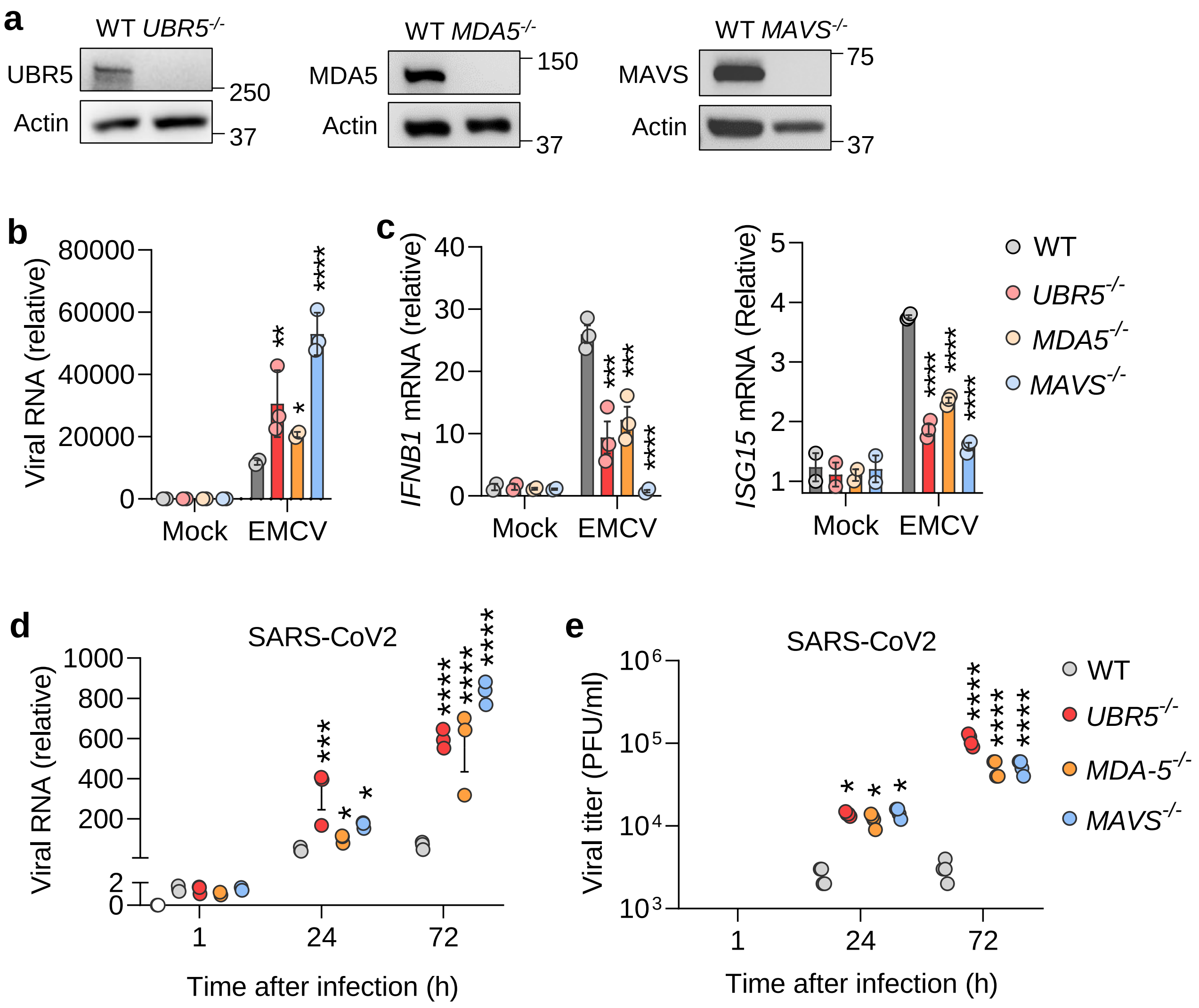

Extended Data Fig. 4. UBR5 is essential for the control of SARS-CoV-2 replication. Related to Fig.3. a) The immunoblots show UBR5/MDA5/MAVS knockout in Calu-3 human lung epithelial cells. Quantification of **b**) intracellular EMCV RNA, **c**) *IFNB1* and *ISG15* mRNA expression by qRT-PCR, in Calu-3 cell infected with EMCV (MOI=0.1) for 12 hr. Mock: no virus; *n*=3 biologically independent experiments. Quantification of **d**) intracellular SARS-CoV-2 RNA by qRT-PCR, *n*=3 biologically independent experiments; **e**) extracellular viral titers by a plaque forming assay, *n*=4 biologically independent experiments. Data shown in **b**-**e** are presented as mean ± S.E.M, two-way ANOVA with Dunnett's test, ***p*=0.0067, **p*=0.0491, *****p*<0.0001 (**b**); ****p*=0.0002, ****p*=0.0008, *****p*<0.0001 in sequence for *IFNB1,* *****p*<0.0001 for *ISG15* (**c**). ****p*=0.0005, **p*=0.0477, **p*=0.0261 for 24 hr, *****p*<0.0001 for 72 hr (**d**); **p*=0.0488, **p*=0.0497, **p*=0.0377 in sequence for 24 hr, *****p*<0.0001 for 72 hr (**e**). Multiplicity adjusted *p* values are presented. Source data are provided as a Source Data file.

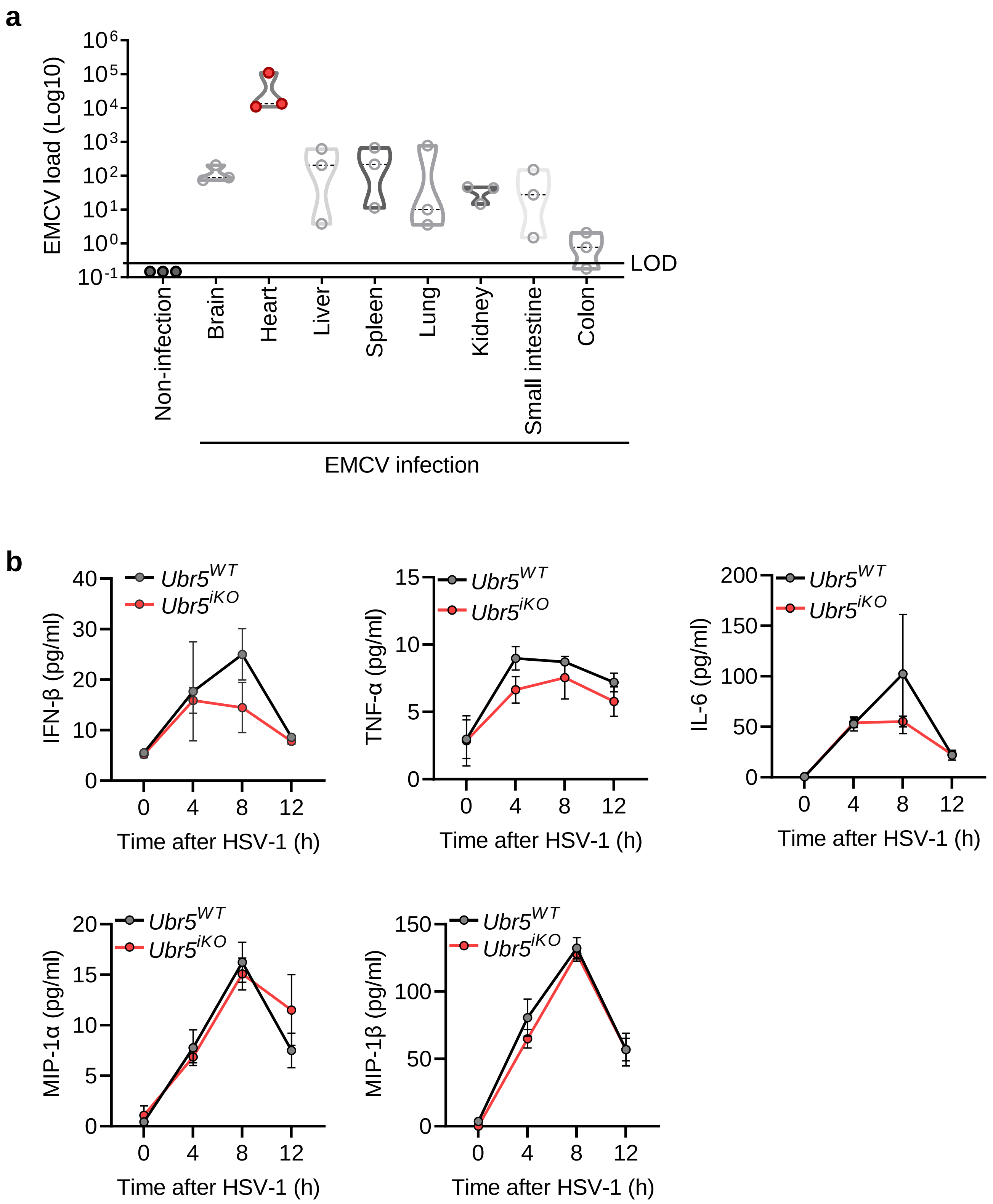

Extended Data Fig. 5. UBR5 is dispensable for the type I IFN response to DNA virus infection in mice. Related to Fig.4. **a)** Quantification of EMCV RNA by qRT-PCR in various tissues of the mice infected with 1,000 PFU of EMCV intraperitoneally for 4 days, *n*=3 mice. **b**) Age- and sex-matched littermates were infected with $1x10^6$ PFU of HSV-1 intravenously. The serum cytokines were quantified by a LegendPlex ELISA. Data shown in **b** are presented as mean ± S.E.M, *n*=2 biologically independent experiments. Source data are provided as a Source Data file.

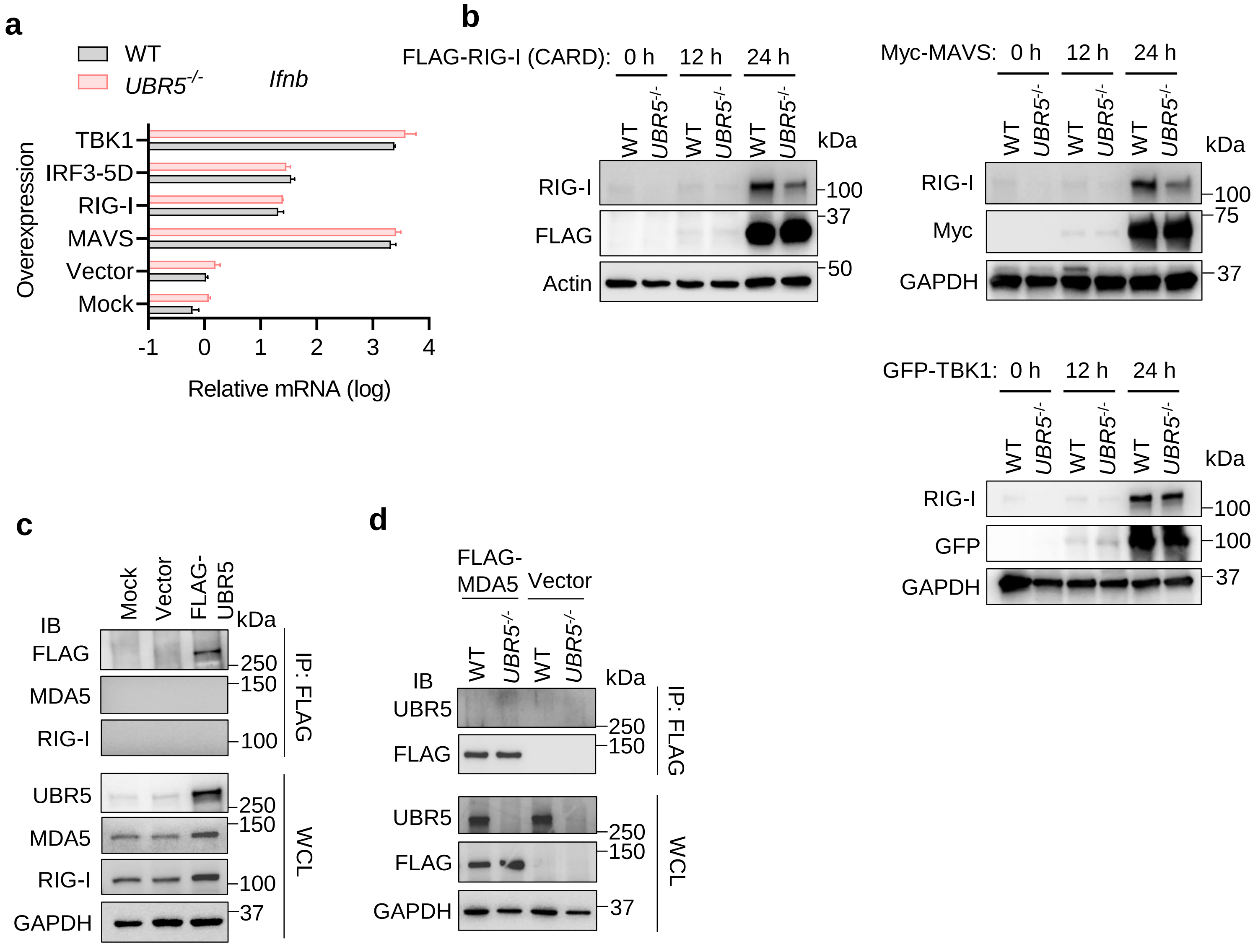

Extended Data Fig. 6. UBR5 does not directly regulate RLR signaling. Related to Fig.5. a) Quantification of *IFNB1* mRNA expression by qRT-PCR, in HEK293 cells transfected with the indicated expression plasmid for 24 hr. IRF3-5D: a mutant IRF3 where the serine or threonine residues at positions 396, 398, 402, 404, and 405 are replaced by phosphomimetic aspartic acid. It is constitutively active to induce type I IFN transcription. Data shown in (**a**) are presented as mean ± S.E.M, two-tailed student's *t*-test, p=0.8123 for Vector, p=0.9046 for MAVS, p=0.9046 for RIG-I, p=0.9049 for IRF3-5D, p=0.7139 for TBK1, n=3 biologically independent experiments. Adjusted p values are presented. **b**) WT and *UBR5*$^{-/-}$ HEK293 cells were transfected with expression plasmids of FLAG-RIGI-I (CARD), Myc-MAVS and GFP-TBK1, respectively, as indicated time course. The endogenous RIG-I expression was then examined by immunoblots as indicated. The data are representative of two independent experiments with similar results. **c**) HEK293 cells were transfected with a FLAG-UBR5 expression or vector plasmid, **d**) HEK293 cells were transfected with a FLAG-MDA5 expression or vector plasmid, for 24 hr. Immunoprecipitation (IP) was performed with an anti-FLAG antibody. The IP and whole cell lysates (WCL) were immunoblotted (IB) with the indicated antibodies. Mock: Lipofectamine only. No UBR5-RLR binding was detected. Source data are provided as a Source Data file.

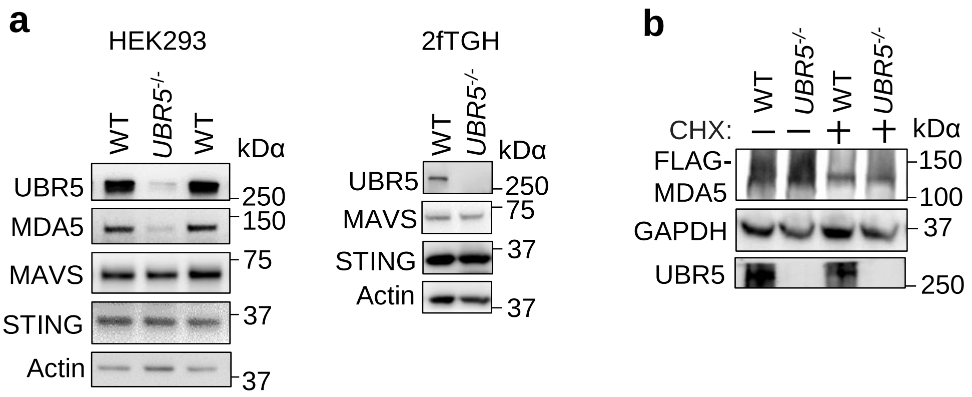

Extended Data Fig. 7. UBR5 regulates RLR expression but not stability. **Related to Fig.5. a)** The immunoblots of indicated protein expression in HEK293 and 2fTGH cells. **b)** Analysis of recombinant MDA5 protein stability by a cycloheximide (CHX) chase assay. A FLAG-MDA5 expression plasmid was transfected into HEK293 cells. At 24 hr after transfection, the cells were treated with 100 µM of CHX (+), or mock-treated (-) for 6 hrs. Whole cell lysates were then subjected to immunoblotting. The data are representative of two independent experiments with similar results.

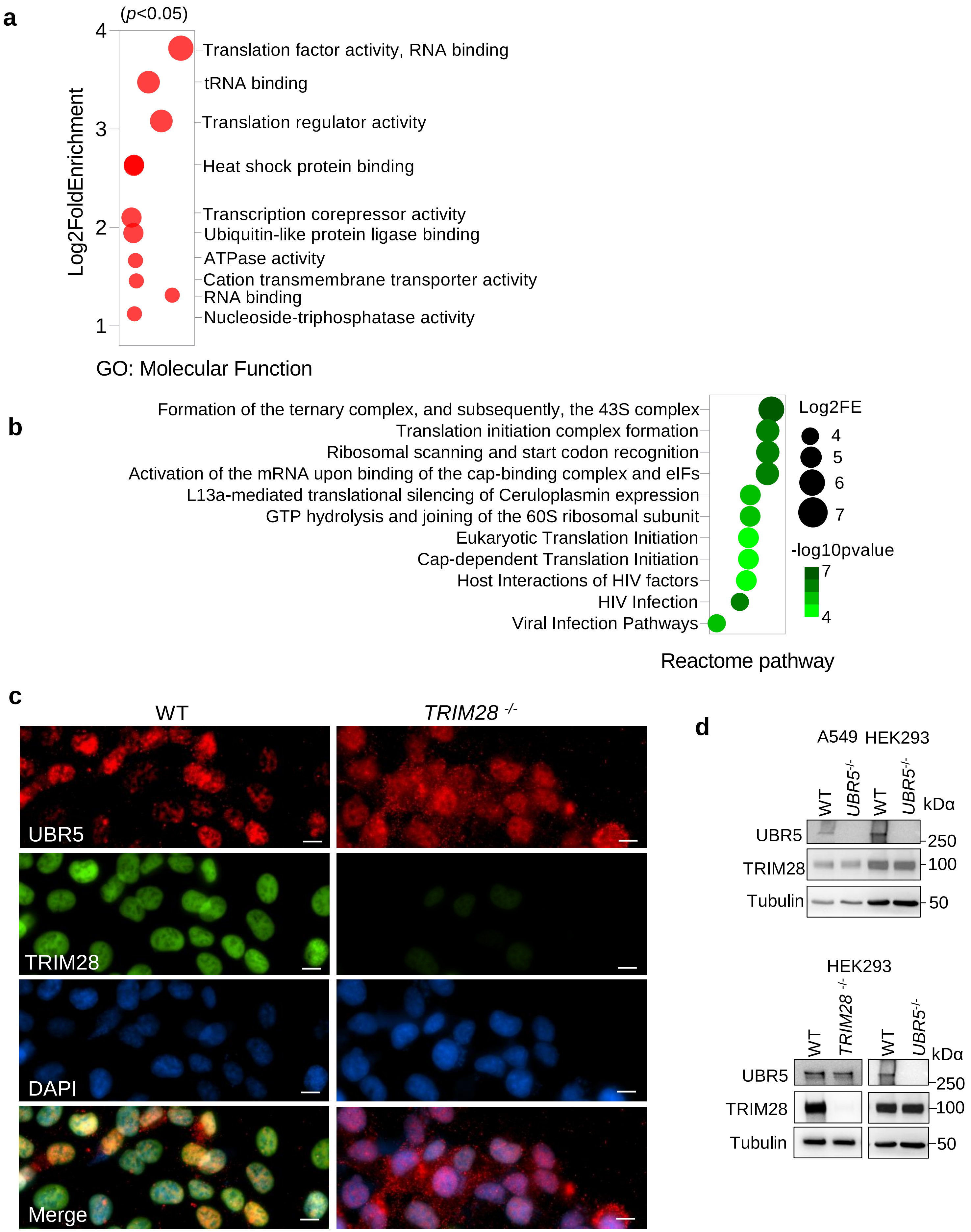

Extended Data Fig. 8. UBR5 is dispensable for maintainingTRIM28 stability and vice versa. Related to Fig.6/7. a, b) GO Molecular function and Reactome pathway enrichment analysis of genes related to interactor of UBR5 identified by IP-UPLC-MS/MS. Log2FE: log2Fold enrichment (*p*<0.05, right-tailed Fisher's exact *t* test with Benjamini & Hochberg). **c**) Immunofluorescent staining of UBR5 and TRIM28 in 2fTGH cells. 4′,6-diamidino-2-phenylindole (DAPI) is a DNA stain. Scale bar: 20 μm. **d)** The immunoblots of UBR5 and TRIM28 proteins in A549 and HEK293 cells. The data are representative of two independent experiments with similar results (**d**).

a

Ub mutants							
6	11	27	29	33	48	63	
K	K	K	K	K	K	K	HA-WTUb
R	R	R	R	R	K	R	HA-K48Ub
R	R	R	R	R	R	K	HA-K63Ub

b

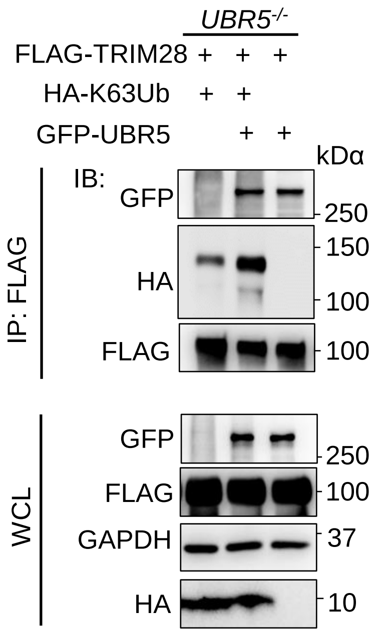

Extended Data Fig. 9. Epichromosomal complementation of UBR5 restores normal K63-linked ubiquitination of TRIM28 in the *UBR5*$^{-/-}$ cells. Related to Fig.7. a) A schematic illustration of HA-WT Ub and its specific K63, K48 ubiquitin mutants. **b)** *UBR5*$^{-/-}$ HEK293 cells were transfected with a FLAG-TRIM28, HA- K63-Ub and GFP-UBR5 plasmids for 24 hr. FLAG-TRIM28 was immunoprecipitated (IP) with an anti-FLAG antibody, and the IP and whole cell lysate (WCL) were immuno-blotted (IB) for the indicated proteins with specific antibodies.

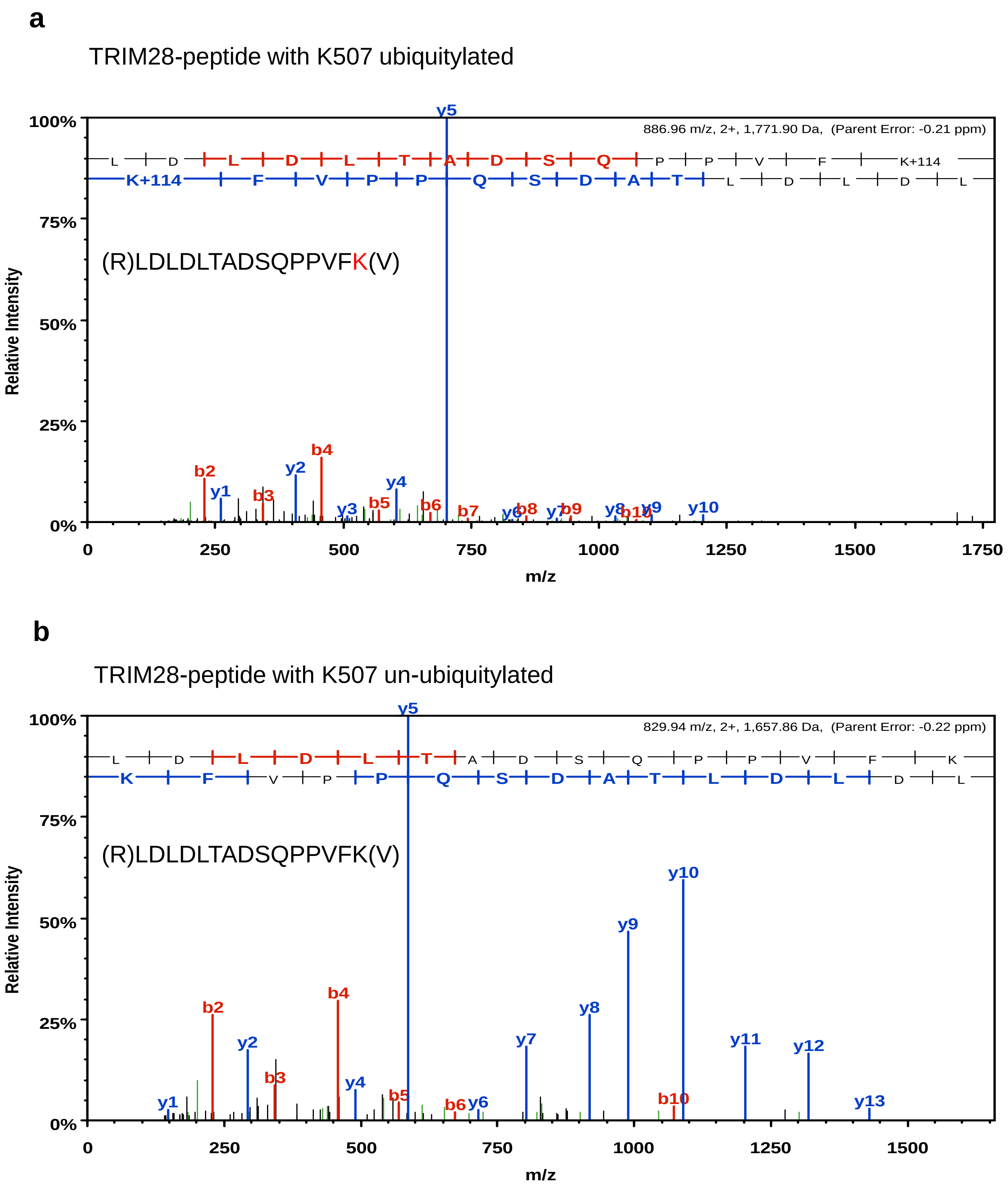

Extended Data Fig. 10. Identification of K507 ubiquitylation site of TRIM28. Related to Fig.7. f, g, h). a) The peptide of LDLDLTADSQPPVFK containing ubiquitylated lysine 507 (in red) of TRIM28 identified by UPLC-MS/MS. **b)** The peptide LDLDLTADSQPPVFK in which the K507 is not ubiquitylated.

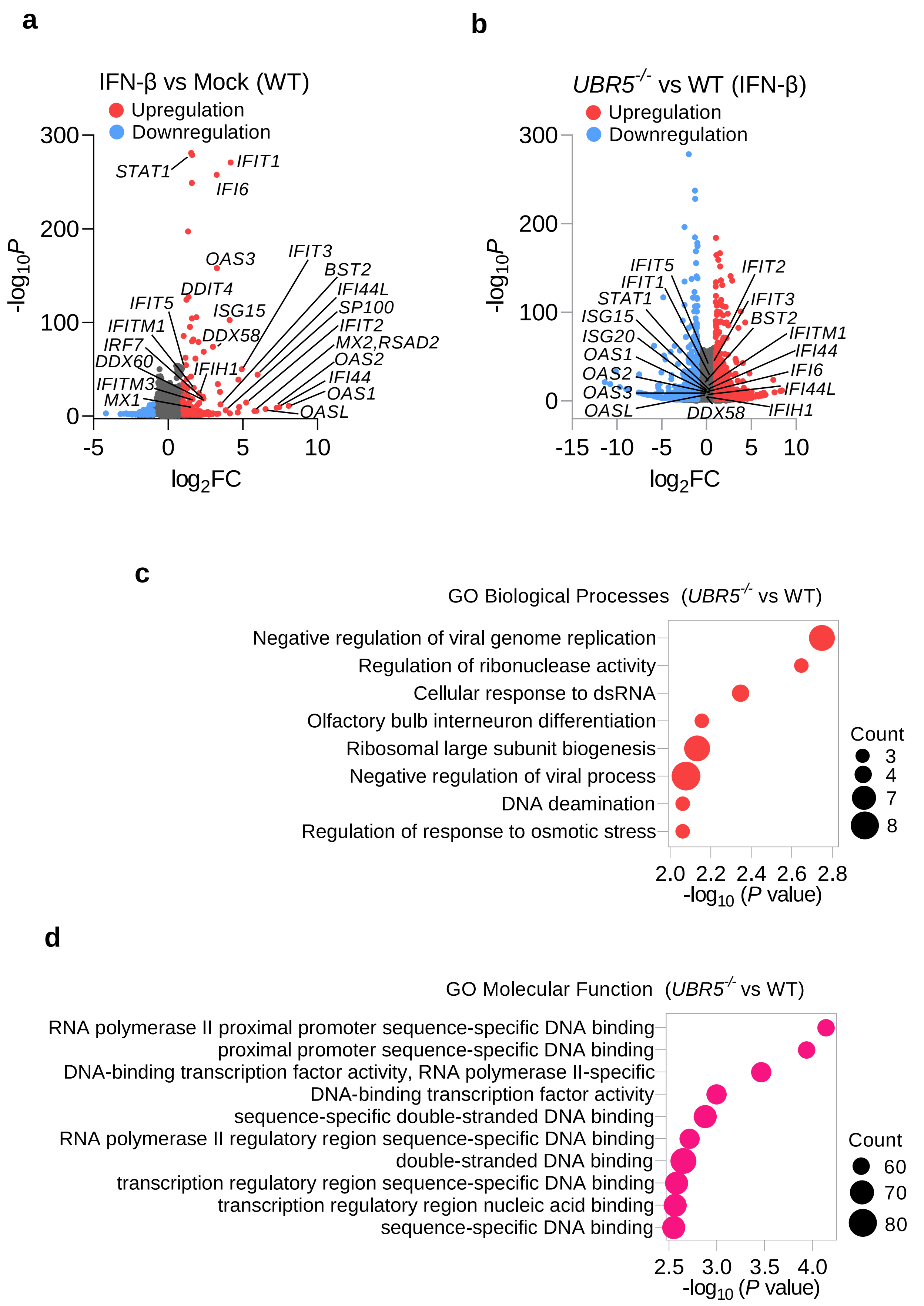

Extended Data Fig. 11. Transcriptome analysis. Related to Fig. 8) Volcano plot of genes differentially expressed in **a**) WT HEK293T cells according to RNA-seq data of IFN-β-treated cells compared to untreated, **b**) IFN-β-treated *UBR5*$^{-/-}$ vs WT. The gray dots represent genes with no statistical difference (-1<Log2FC<1). Red dots: Log2FC>1, p <0.05. Blue dots: Log2FC<-1, p <0.05, the Wald test was used to generate log2FC and p-values adjusted with the Benjamini-Hochberg. **c, d**) Gene ontology analysis of differentially expressed gens in poly (I:C)-treated *UBR5*$^{-/-}$ cells vs WT (p<0.05, right-tailed Fisher's exact t test with Benjamini & Hochberg).

UBR5 promotes antiviral immunity by disengaging the transcriptional brake on RIG-I like receptors

Duomeng Yang, Tingting Geng, Andrew G. Harrison, Jason G Cahoon, Jian Xing, Baihai Jiao, Mark Wang, Chao Cheng, Robert E. Hill, Huadong Wang, Anthony T. Vella, Gong Cheng, Yanlin Wang, Penghua Wang

Uncropped blots

Uncropped blots for Fig.2

Fig.2 a

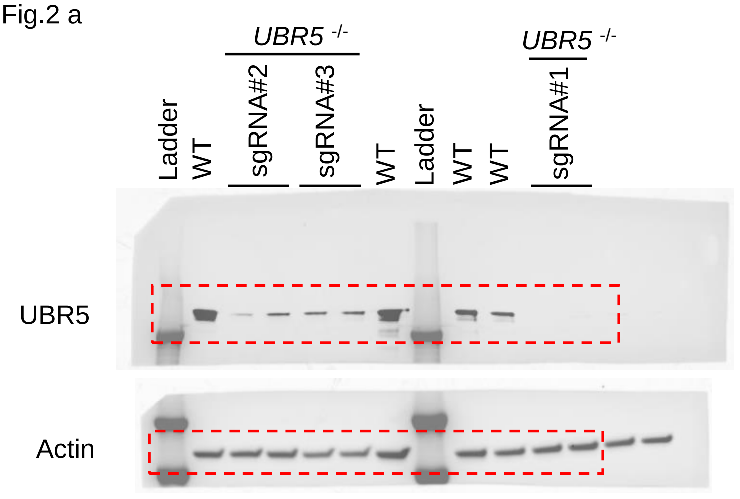

Fig.2 l

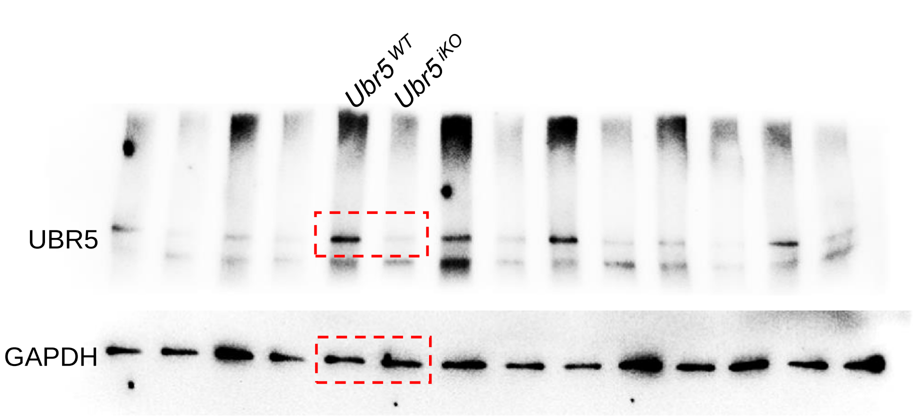

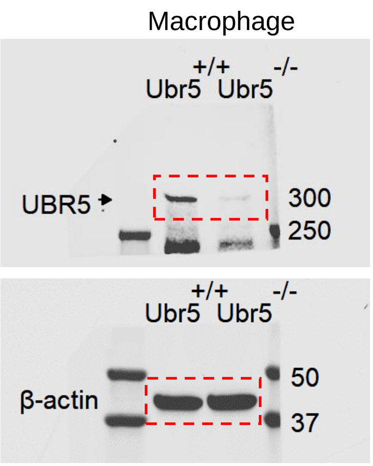

Uncropped blots for Fig.3

Fig.3e

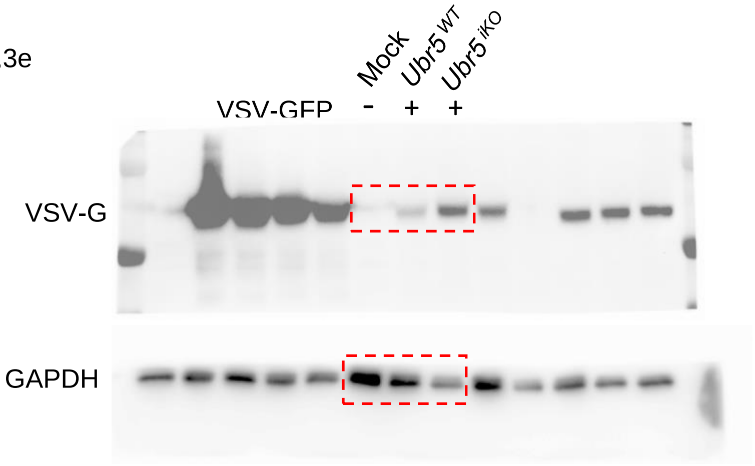

Uncropped blots for Fig.4

Fig.4 a

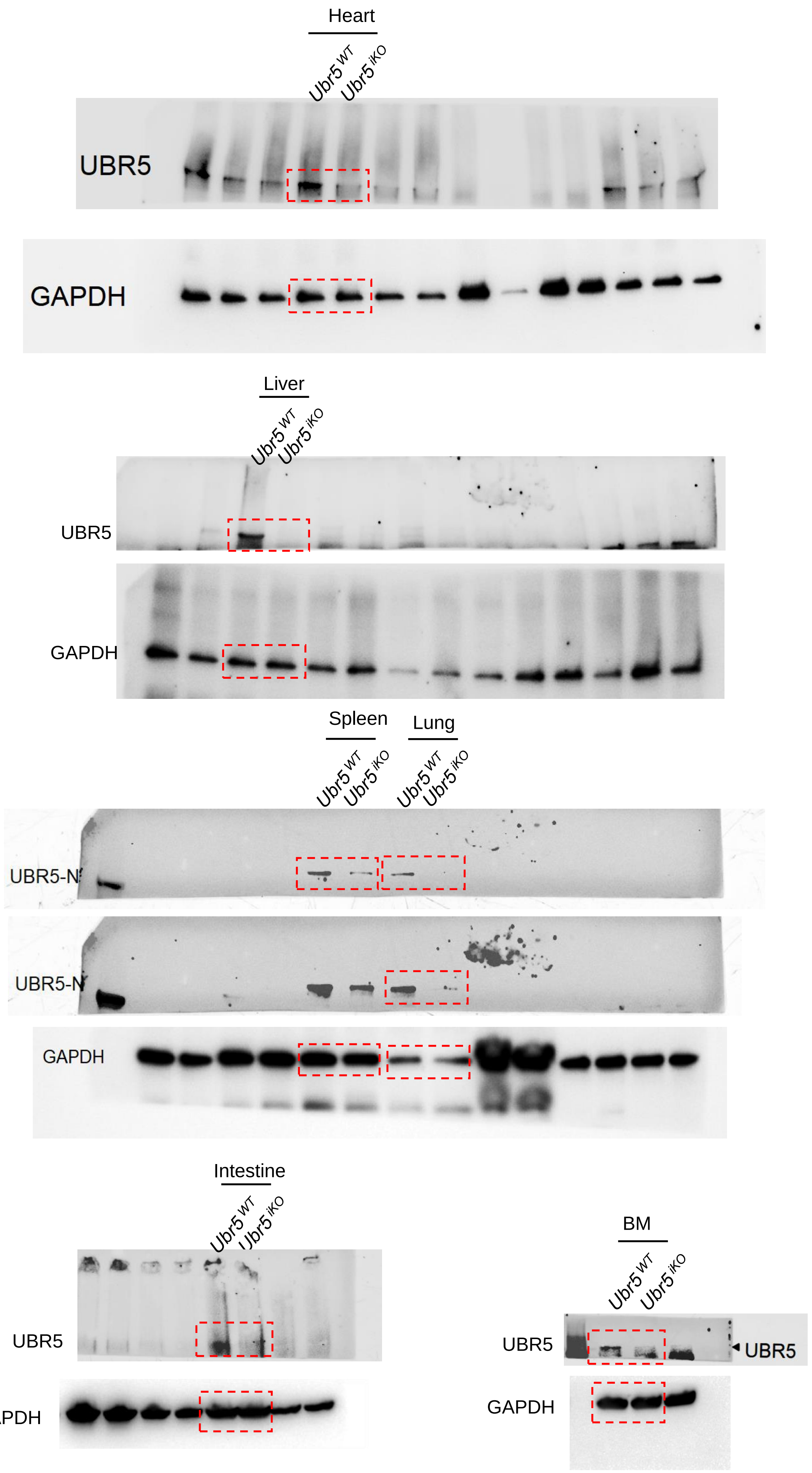

Uncropped blots for Fig.5

Fig.5a

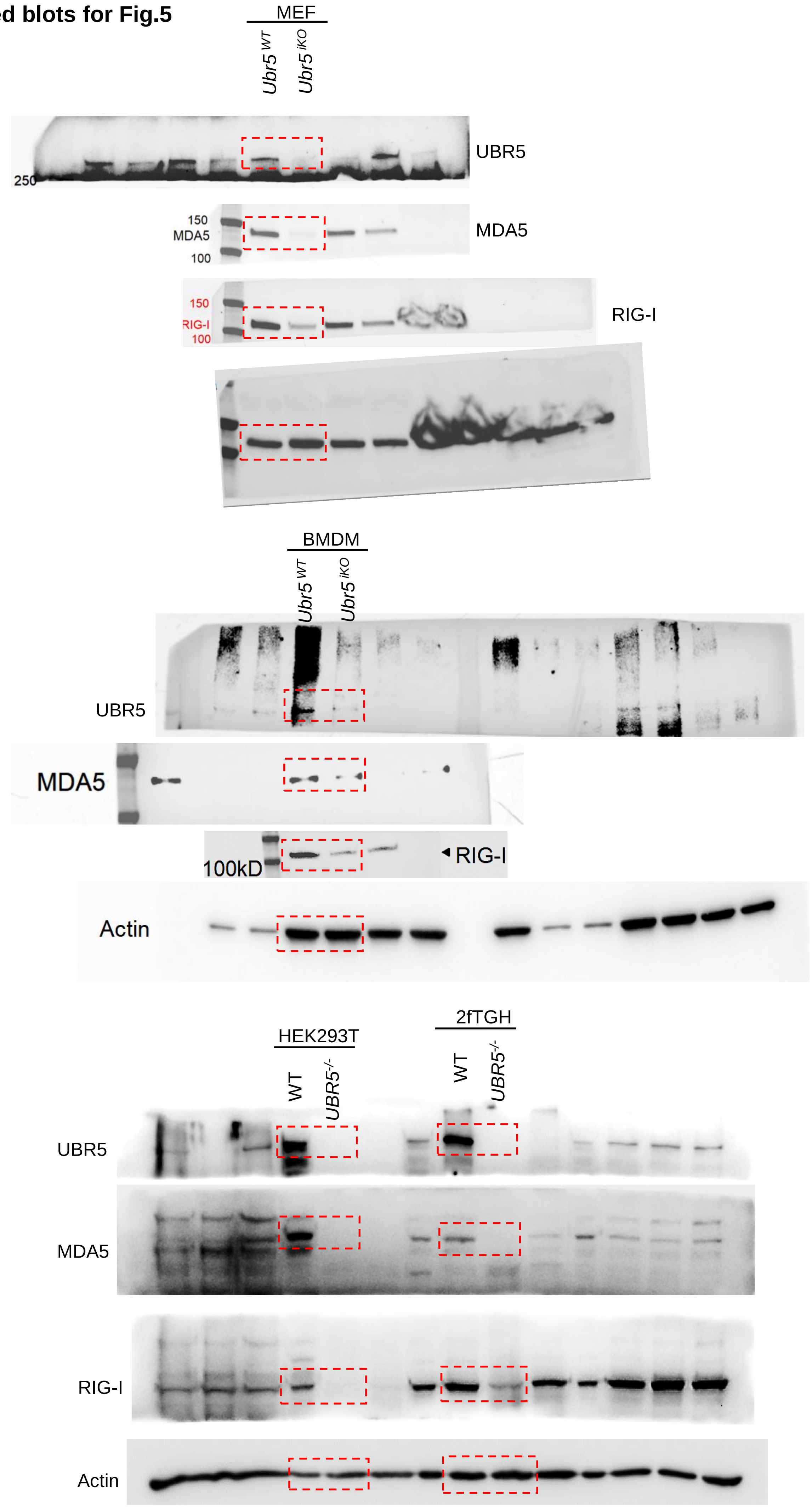

Uncropped blots for Fig.5

Fig.5a continue

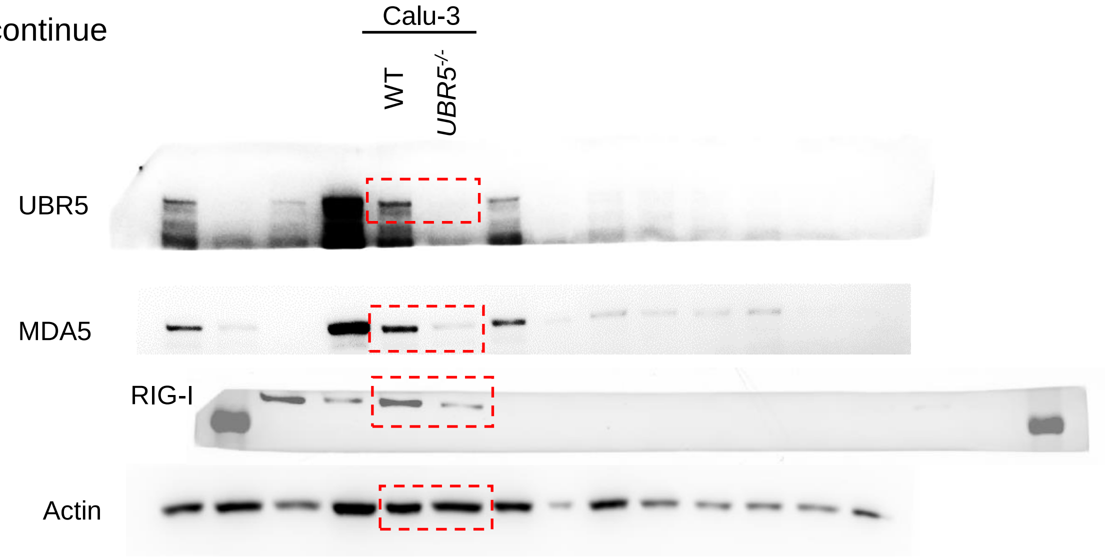

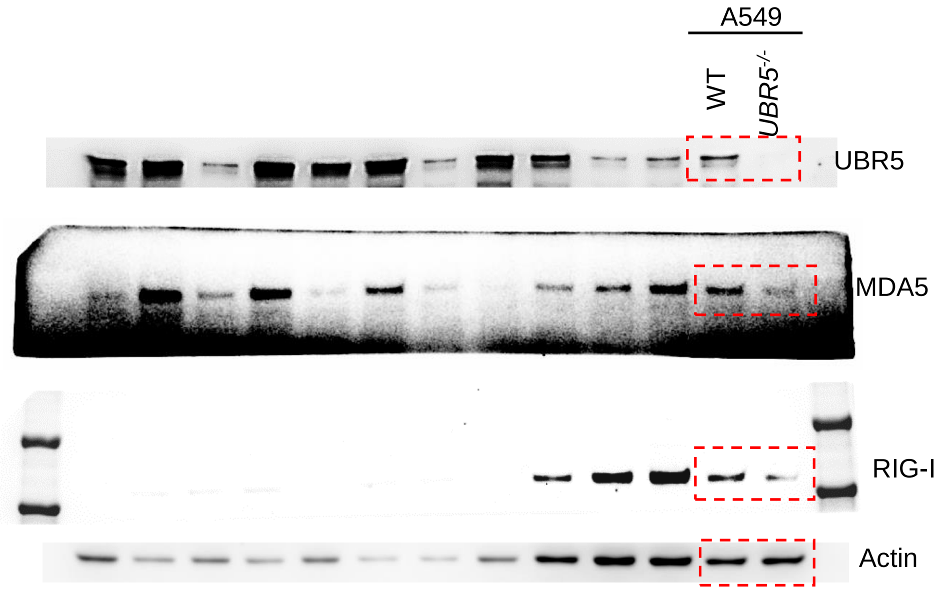

Fig.5b

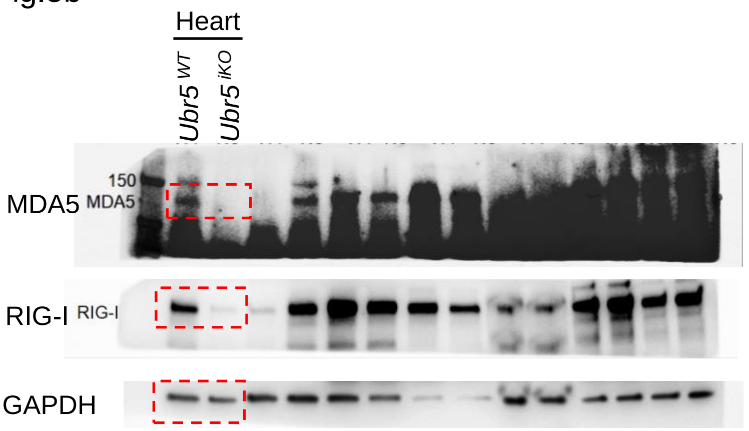

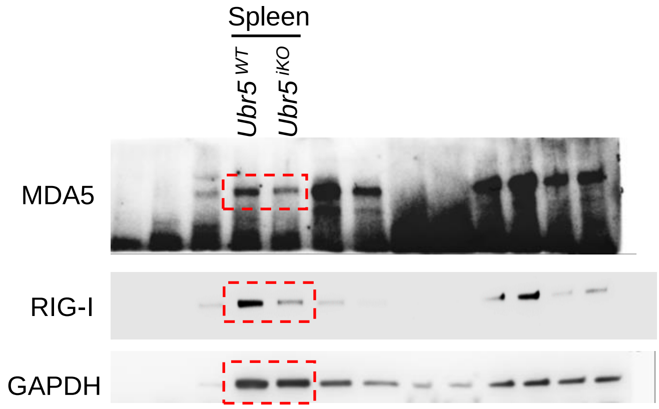

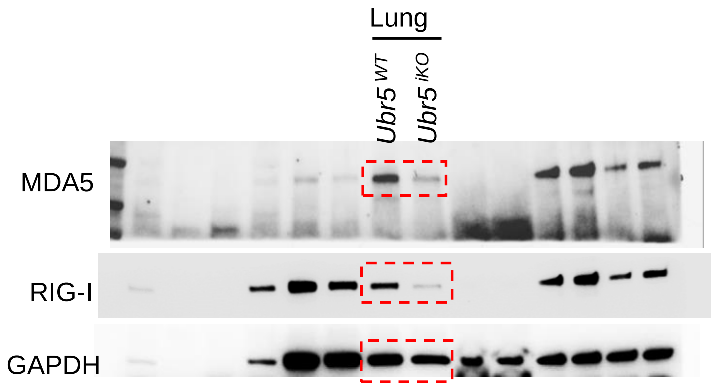

Uncropped blots for Fig.5

Fig.5c

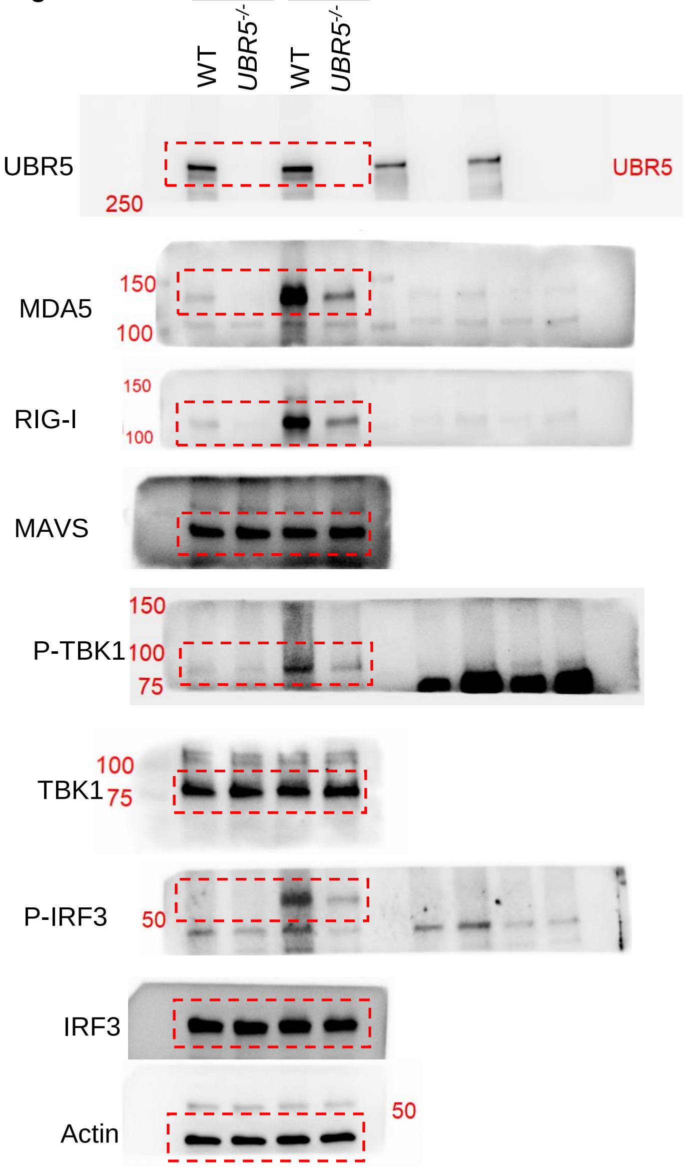

Fig.5d

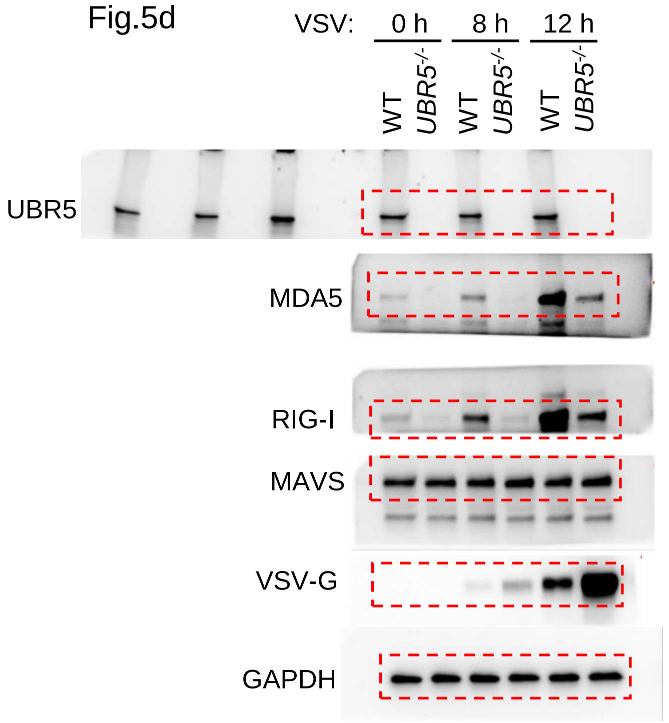

Fig.5e

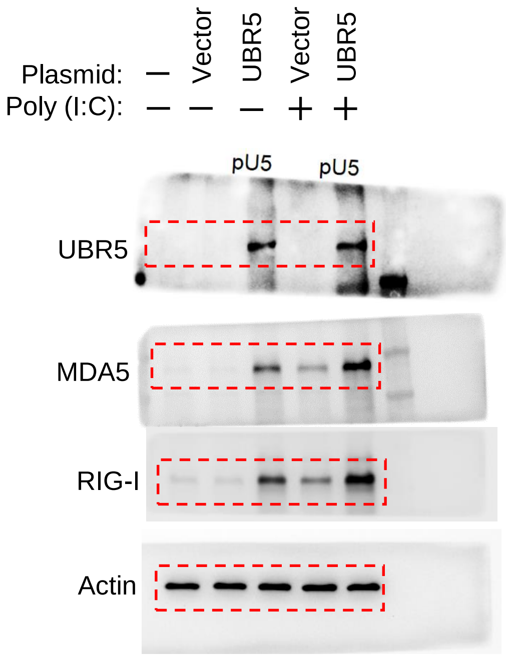

Fig.5f

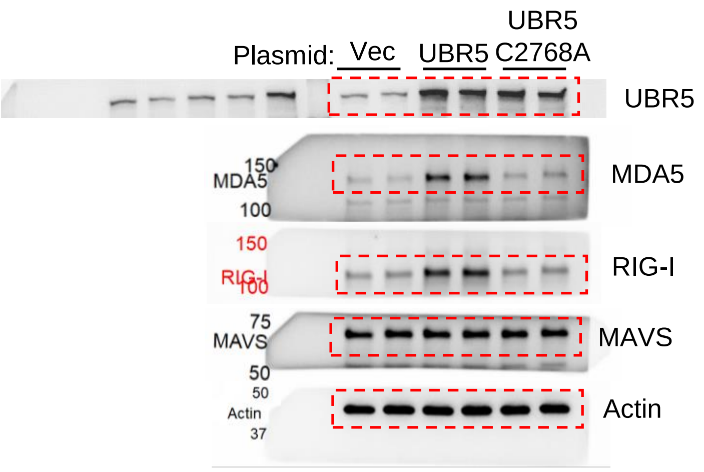

Uncropped blots for Fig.6

Fig. 6a

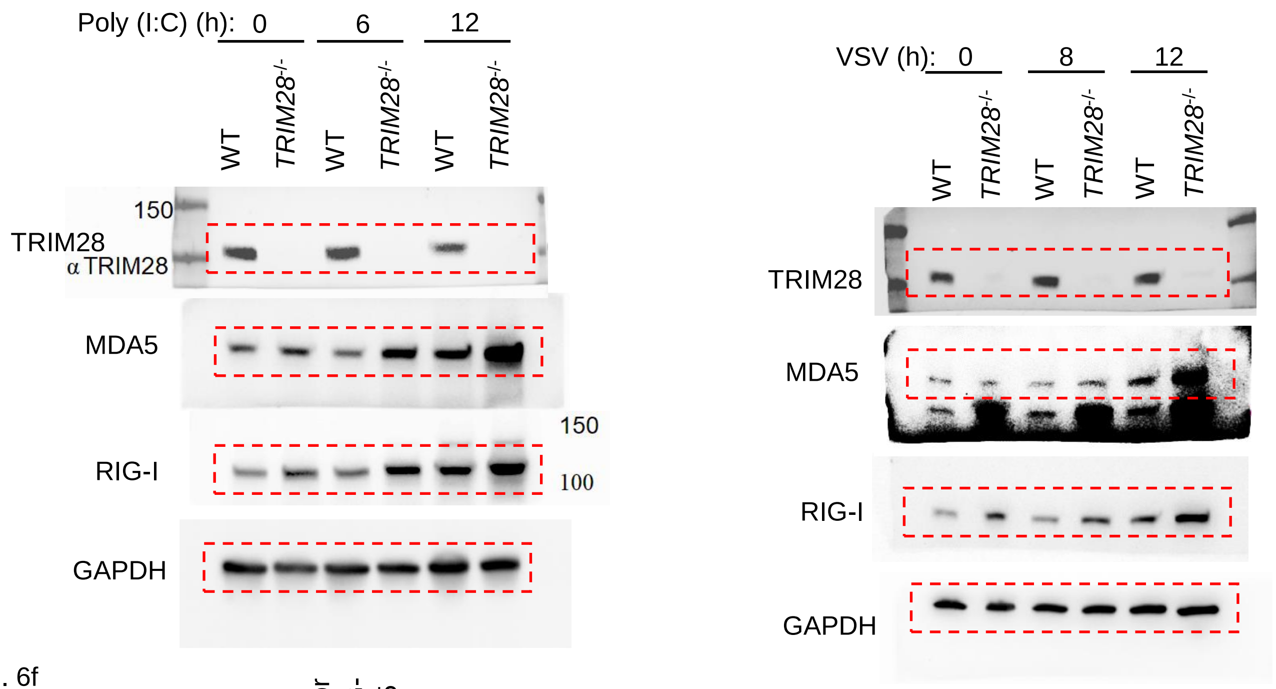

Fig. 6f

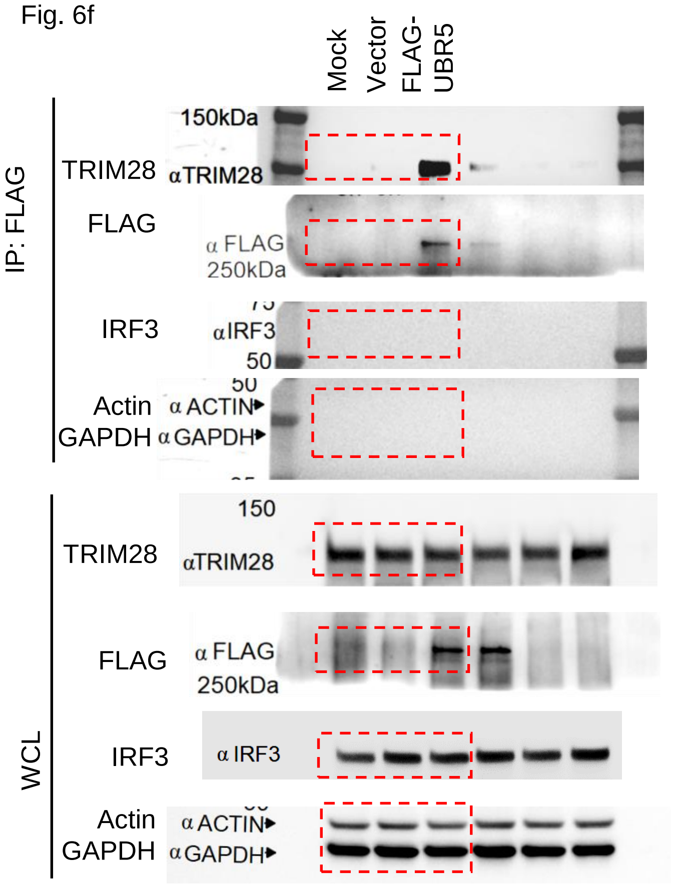

Fig. 6g

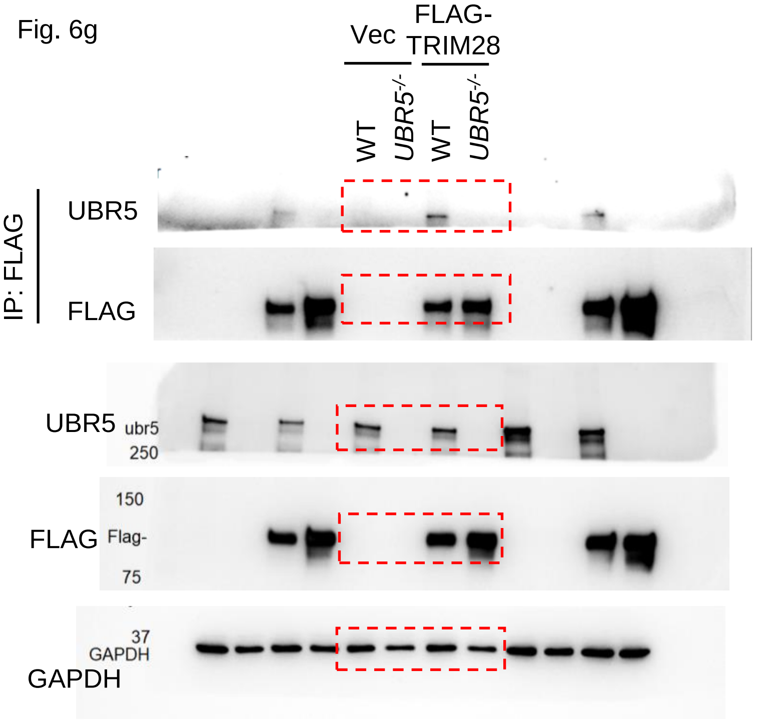

Fig. 6h

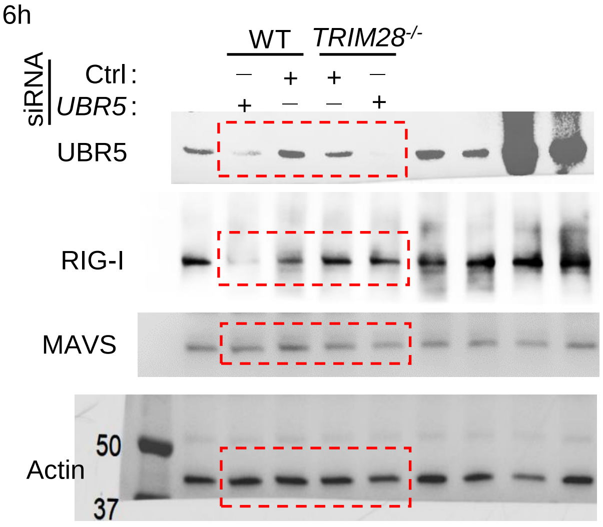

Fig. 6i

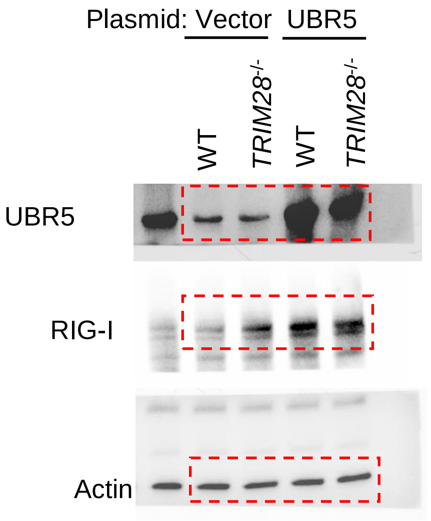

Uncropped blots for Fig.7

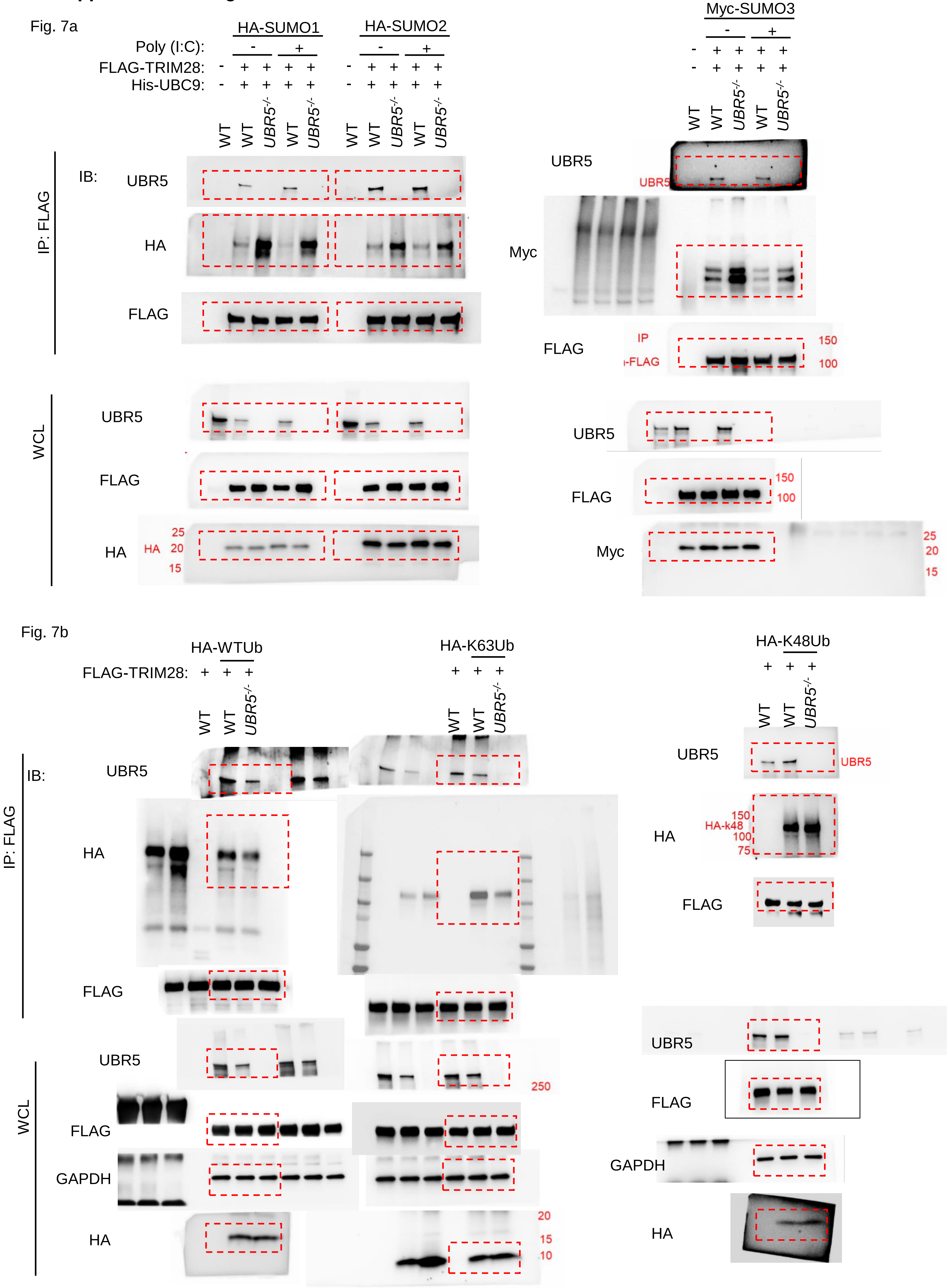

Uncropped blots for Fig.7

Fig. 7c

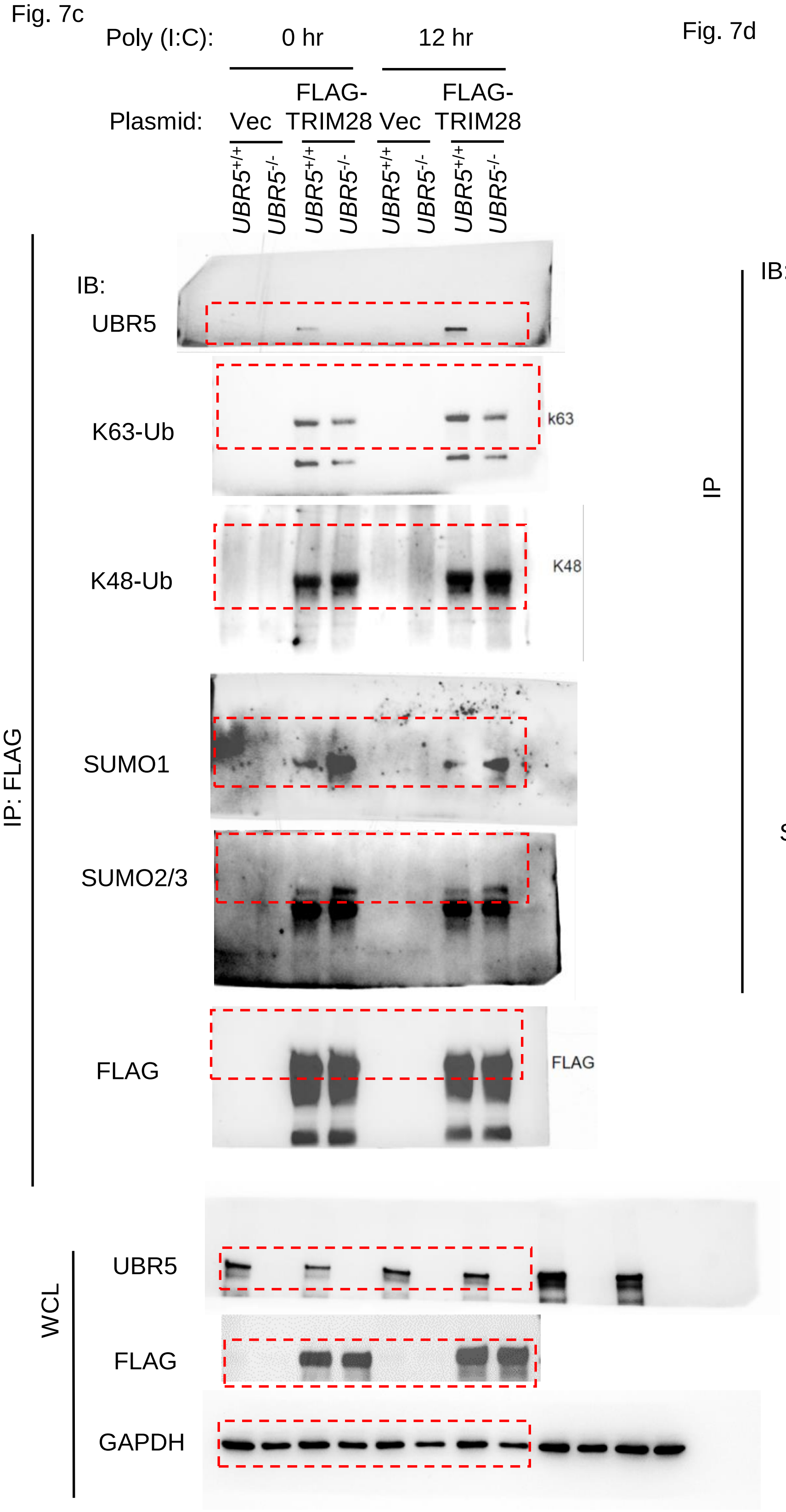

Fig. 7d

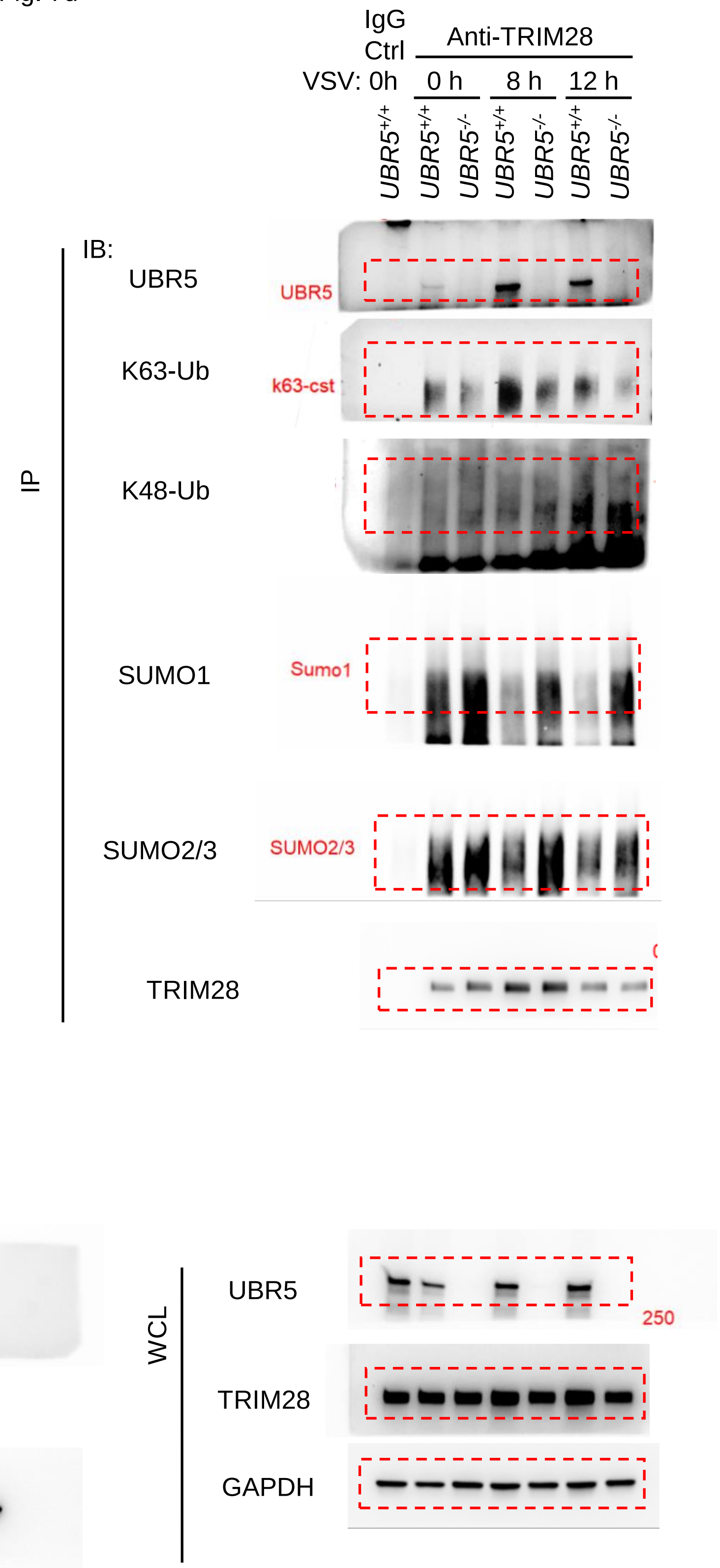

Uncropped blots for Fig.7

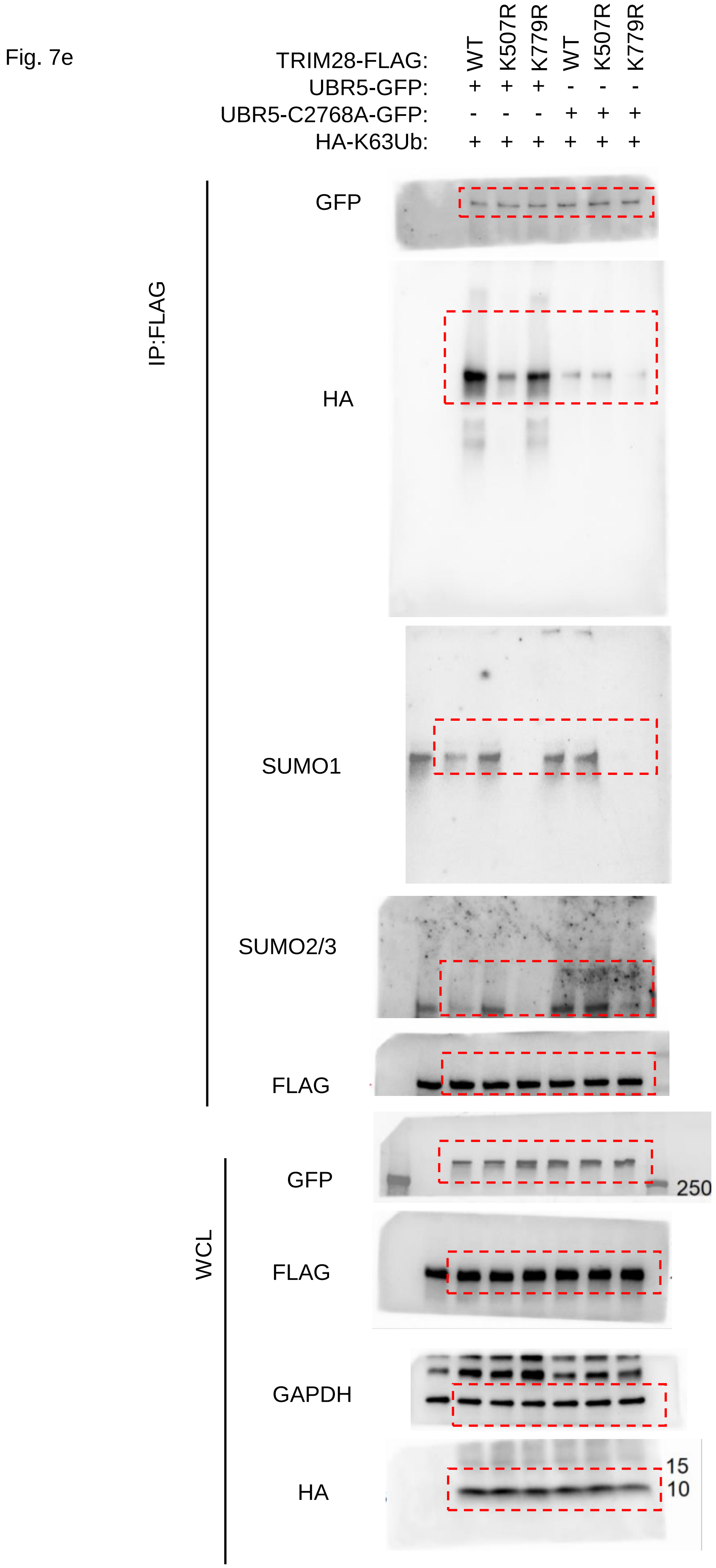

Uncropped blots for Extended Data Figures

Extended Data Fig. 1.d

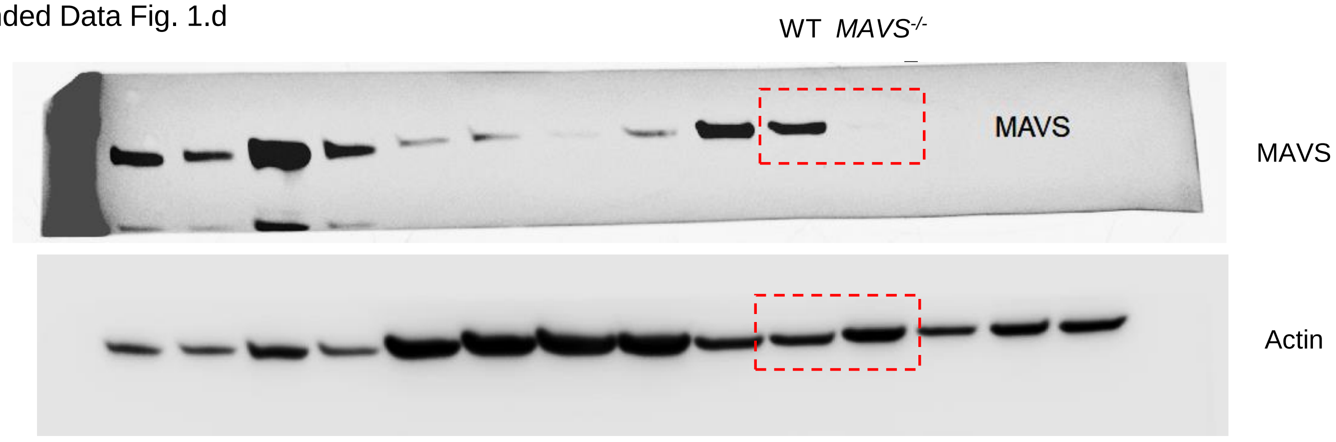

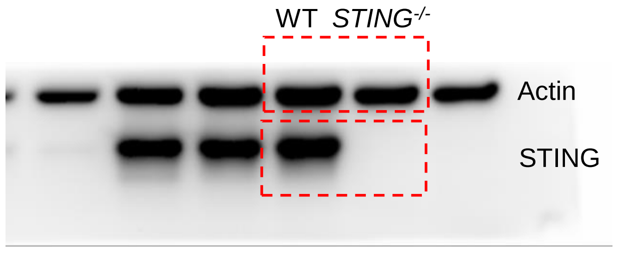

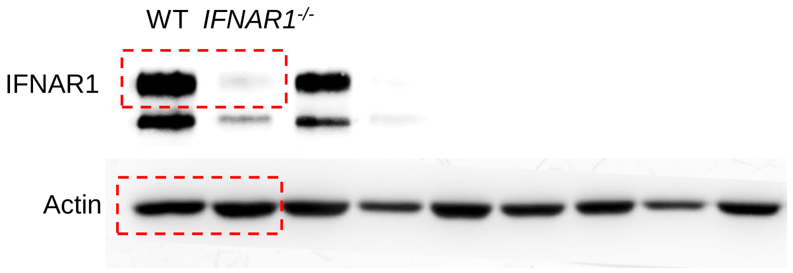

Extended Data Fig. 3.g

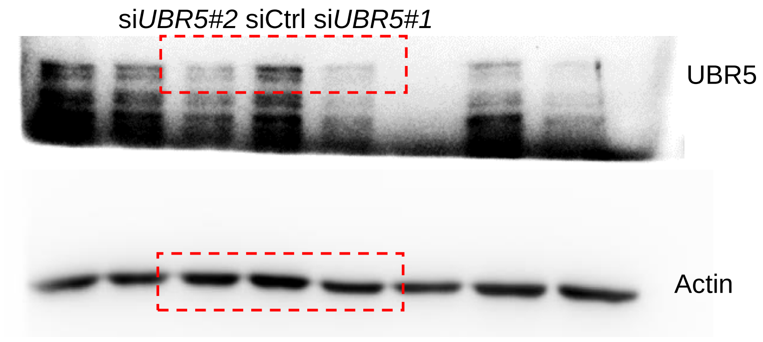

Extended Data Fig. 4.a

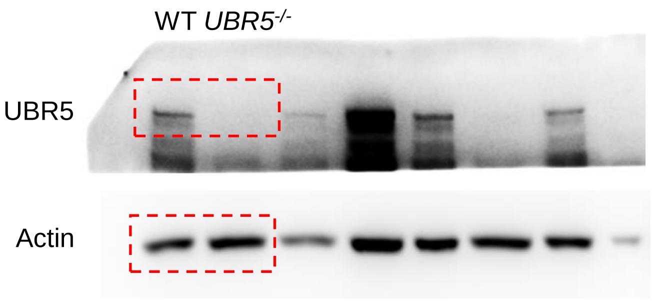

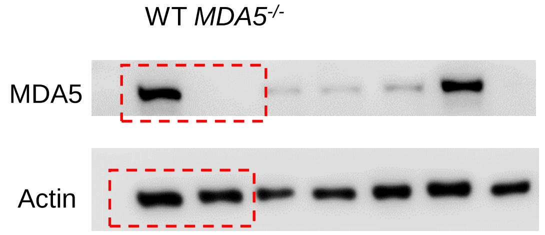

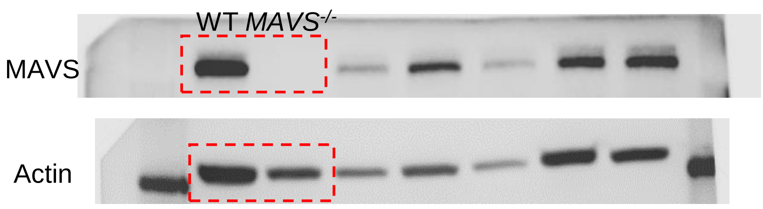

Uncropped blots for Extended Data Figures

Extended Data Fig. 6.b

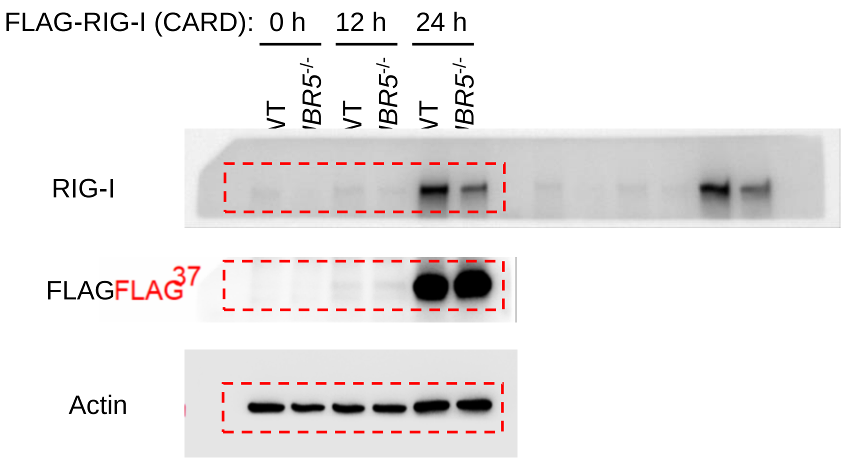

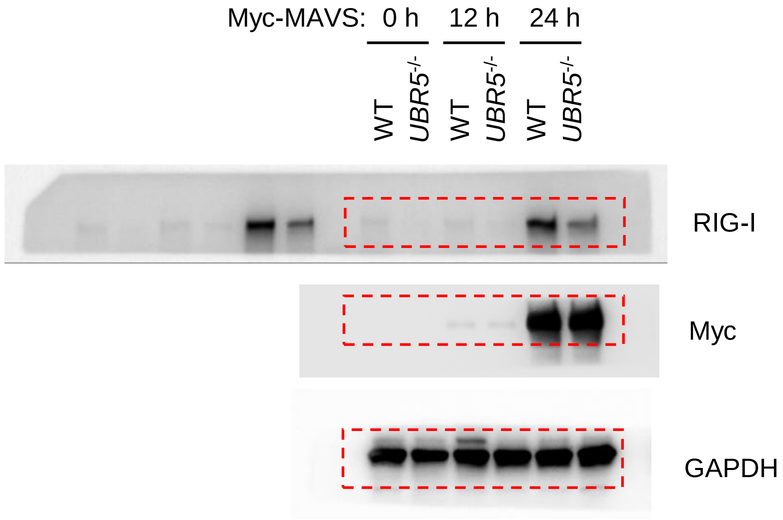

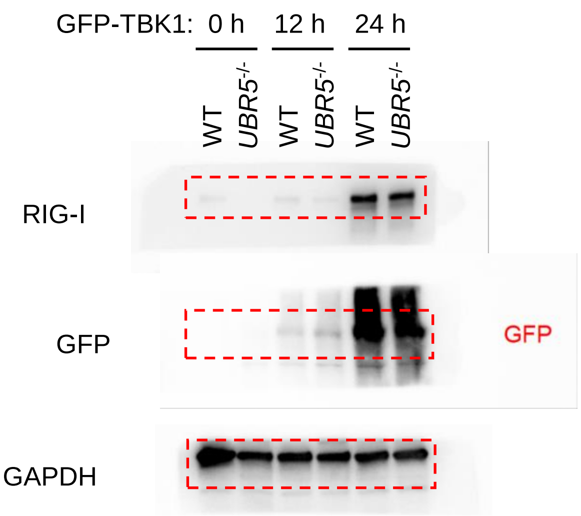

Uncropped blots for Extended Data Figures

Extended Data Fig. 6.c

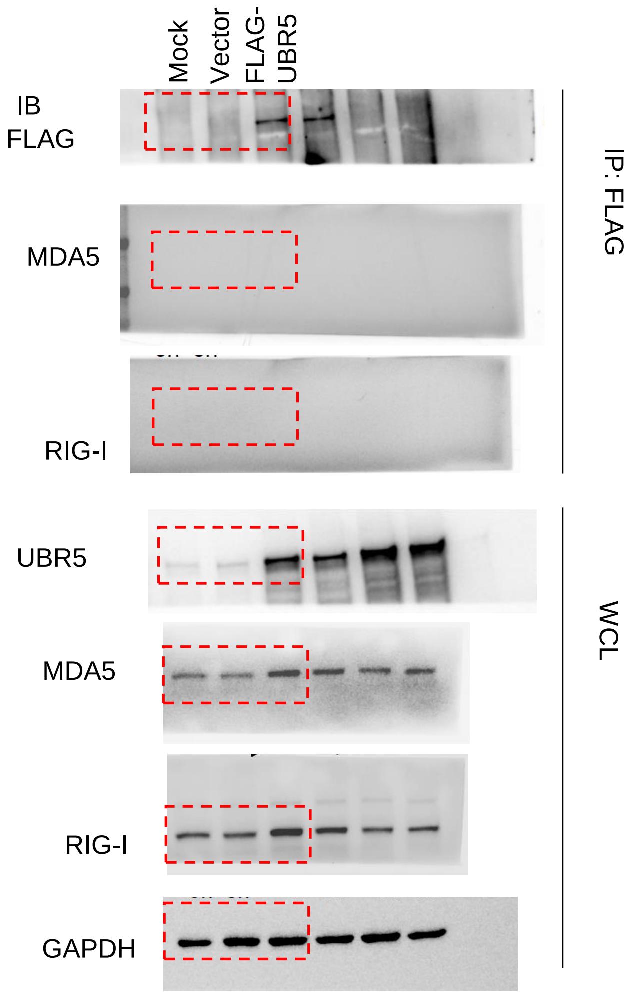

Extended Data Fig. 6.d

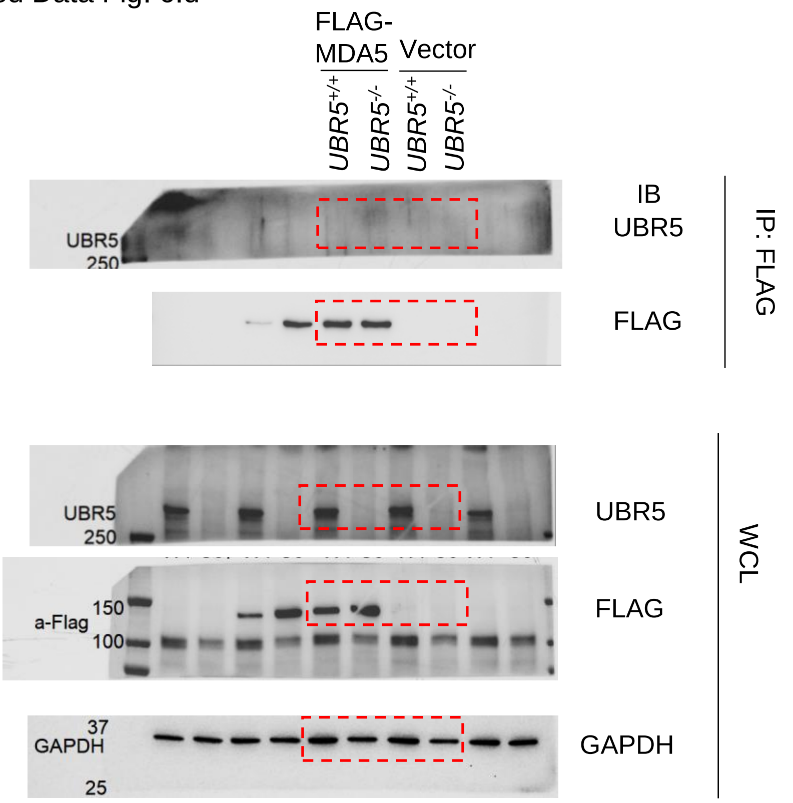

Extended Data Fig. 7.a

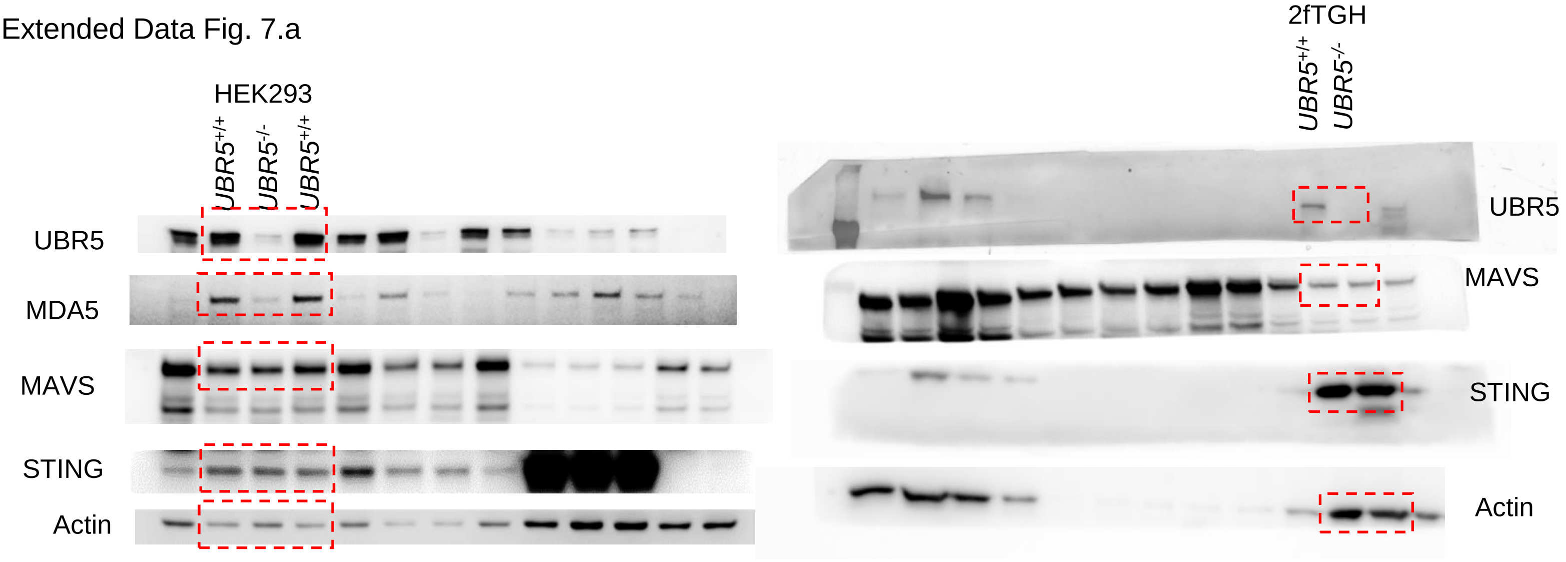

Extended Data Fig. 7.b

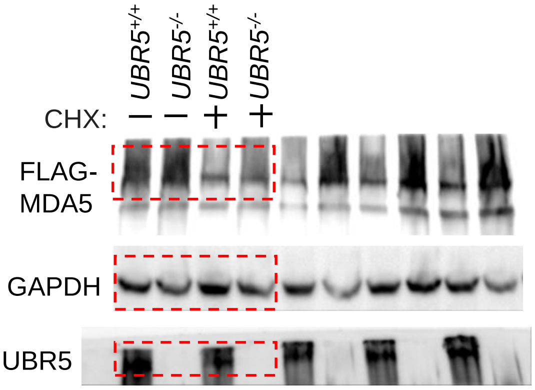

Uncropped blots for Extended Data Figures

Extended Data Fig. 8.b

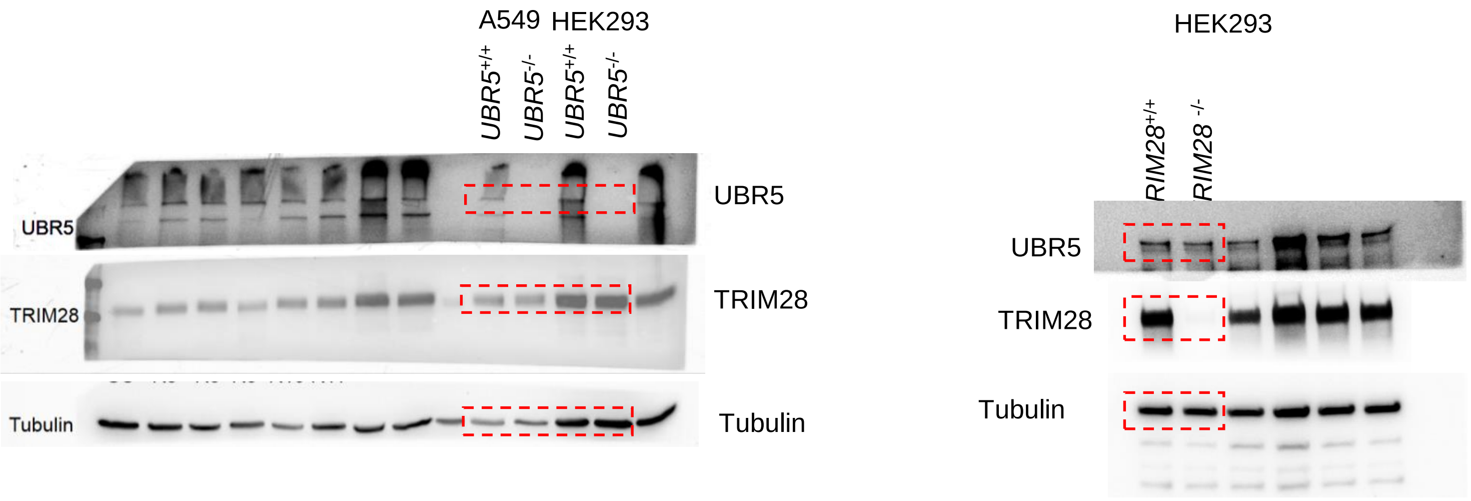

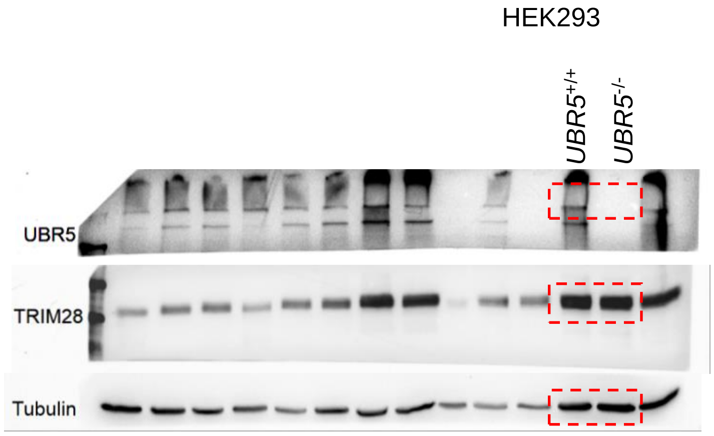

Extended Data Fig. 9.b

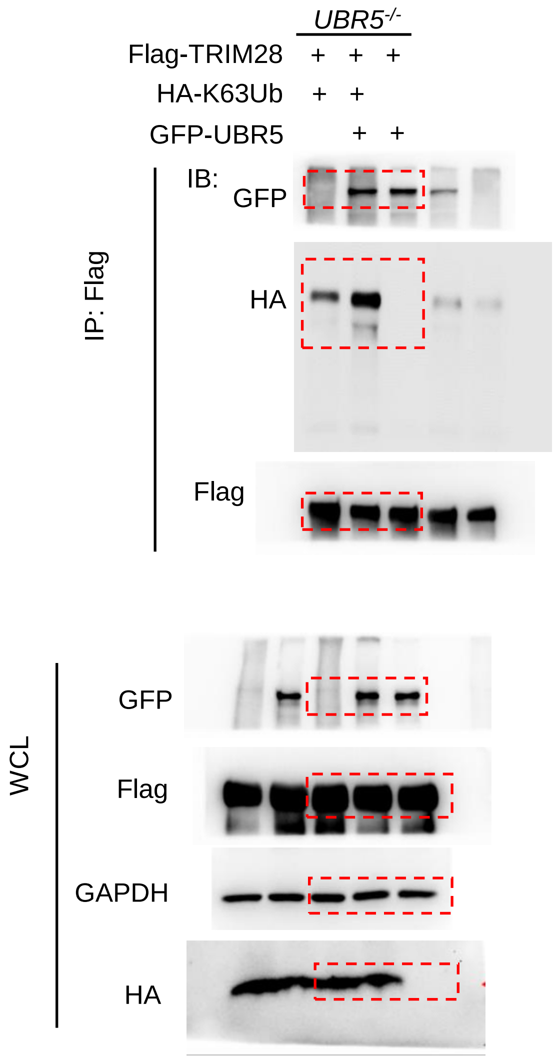

Supplement: Supplementary file 1 — Supplementary Information [file 41467_2024_45141_MOESM1_ESM.pdf]
