# Supplementary material for: UBR5 promotes antiviral immunity by disengaging the transcriptional brake on RIG-I like receptors
Source: Nat Commun. 2024 Jan 26;15:780. doi: 10.1038/s41467-024-45141-1 (PMC10817939; doi:10.1038/s41467-024-45141-1)
Supplement: Supplementary file 3 — Description of Additional Supplementary Files [file 41467_2024_45141_MOESM3_ESM.pdf]

Description of Additional Supplementary Files

Supplementary Data 1. The CRISPR-Cas9 guide RNA sequences for individual E3 ligases. Related to Fig.1 and Methods.

Supplementary Data 2. The fold changes in the ISRE-Luc activity of individual E3 knockout lines relative to wild type. Related to Fig.1.

Supplementary Data 3. The CRISPR-Cas9 guide RNA sequences for additional human genes. Related to Fig.1/2 and Methods.

Supplementary Data 4. The oligo DNA sequences of select E3 genes for the T7 endonuclease I (T7EI) mismatch cleavage-assay. Related to Fig.1 and Methods.

Supplementary Data 5. Interactors of UBR5 identified by UPLC-MS/MS analysis. Related to Fig.6.

Supplementary Data 6. The ubiquitinated lysine residues of TRIM28 and modification frequency in *UBR5*$^{-/-}$ and WT cells identified by UPLC-MS/MS. Related to Fig.7.

Supplementary Data 7. Primers for the Site-direct mutagenesis of TRIM28 and sequencing. Related to Fig.7 and methods.

Supplementary Data 8. ChIP-seq identification of gene promoters significantly enriched by TRIM28 in *UBR5*$^{-/-}$ versus WT HEK293T cells with VSV infection. The *p* values were generated by two-tailed student's *t*-test. Related to Fig.7.

Supplementary Data 9. Downregulated and upregulated DEGs in *UBR5*$^{-/-}$ and *TRIM28*$^{-/-}$ vs WT cells with/or without Poly (I:C) stimulation. The Wald test was used to generate log2FC and *p*-values adjusted with the Benjamini-Hochberg. The right-tailed Fisher's exact *t* test with Benjamini & Hochberg was used in pathway analysis. Related to Fig.8 and Extended Data Fig. 11.

Supplementary Data 10. DEGs in *UBR5*$^{-/-}$ versus WT HEK293 cells stimulated with IFN-β. The Wald test was used to generate log2FC and *p*-values adjusted with the Benjamini-Hochberg. Related to Fig. 8 and Extended Data Fig. 11.

Supplementary Data 11. ISGs induced by IFN-β in WT cells and further upregulated by TRIM28 deficiency. The Wald test was used to generate log2FC and *p*-values adjusted with the Benjamini-Hochberg. Related to Fig.8 and Extended Data Fig. 11.
