# Supplementary material for: UBR5 promotes antiviral immunity by disengaging the transcriptional brake on RIG-I like receptors
Source: Nat Commun. 2024 Jan 26;15:780. doi: 10.1038/s41467-024-45141-1 (PMC10817939; doi:10.1038/s41467-024-45141-1)
Supplement: Supplementary file 15 — Reporting Summary [file 41467_2024_45141_MOESM15_ESM.pdf]

Corresponding author(s): Penghua Wang
Last updated by author(s): Dec 23, 2023

Reporting Summary

Nature Portfolio wishes to improve the reproducibility of the work that we publish. This form provides structure for consistency and transparency in reporting. For further information on Nature Portfolio policies, see our Editorial Policies and the Editorial Policy Checklist.

Statistics

For all statistical analyses, confirm that the following items are present in the figure legend, table legend, main text, or Methods section.

n/a	Confirmed	
☐	☒	The exact sample size (*n*) for each experimental group/condition, given as a discrete number and unit of measurement
☐	☒	A statement on whether measurements were taken from distinct samples or whether the same sample was measured repeatedly
☐	☒	The statistical test(s) used AND whether they are one- or two-sided<br>*Only common tests should be described solely by name; describe more complex techniques in the Methods section.*
☒	☐	A description of all covariates tested
☒	☐	A description of any assumptions or corrections, such as tests of normality and adjustment for multiple comparisons
☐	☒	A full description of the statistical parameters including central tendency (e.g. means) or other basic estimates (e.g. regression coefficient) AND variation (e.g. standard deviation) or associated estimates of uncertainty (e.g. confidence intervals)
☐	☒	For null hypothesis testing, the test statistic (e.g. *F*, *t*, *r*) with confidence intervals, effect sizes, degrees of freedom and *P* value noted<br>*Give P values as exact values whenever suitable.*
☒	☐	For Bayesian analysis, information on the choice of priors and Markov chain Monte Carlo settings
☒	☐	For hierarchical and complex designs, identification of the appropriate level for tests and full reporting of outcomes
☒	☐	Estimates of effect sizes (e.g. Cohen's *d*, Pearson's *r*), indicating how they were calculated

Our web collection on statistics for biologists contains articles on many of the points above.

Software and code

Policy information about availability of computer code

Data collection	Real-Time PCR:Bio-RAD CFX96; RNA-seq:the Illumina HiSeq 2500;Confocal images: Zeiss LSM 850+Airyscan2 confocal microscopes.Bead-based multiplex ELISA:BIORAD ZE5; Western blot: Bio-rad ChemiDoc MP Imaging System; ISRE readout: BioTek CITATION Imaging reader.
Data analysis	Statistics and Data plotting: GraphPad Prism 8.0; for RNA-seq: fastp v.0.23.1,UMI-based deduplication was performed using fastp v.0.23.1. Trimmed and deduplicated reads were then mapped to human reference assembly GRCh38 using the STAR aligner v.2.5.2b 59to generate BAM files. Gene hit counts were calculated by using featureCounts from the Subread package v.1.5.2 60. DESeq2 was used to perform differential expression analysis between the defined groups of samples. GelAnalyzer2010a, LEGENDPlex data analysis software.

For manuscripts utilizing custom algorithms or software that are central to the research but not yet described in published literature, software must be made available to editors and reviewers. We strongly encourage code deposition in a community repository (e.g. GitHub). See the Nature Portfolio guidelines for submitting code & software for further information.

Data

Policy information about availability of data

All manuscripts must include a data availability statement. This statement should provide the following information, where applicable:

- Accession codes, unique identifiers, or web links for publicly available datasets
- A description of any restrictions on data availability
- For clinical datasets or third party data, please ensure that the statement adheres to our policy

The datasets generated during and/or analyzed during the current study are in the supplemental materials. Source data are provided with this paper. The raw data

and processed data for RNA-seq and ChIP-seq are available at National Center for Biotechnology Information GEO (GSE245604).

Research involving human participants, their data, or biological material

Policy information about studies with human participants or human data. See also policy information about sex, gender (identity/presentation), and sexual orientation and race, ethnicity and racism.

Reporting on sex and gender	N/A
Reporting on race, ethnicity, or other socially relevant groupings	N/A
Population characteristics	N/A
Recruitment	N/A
Ethics oversight	N/A

Note that full information on the approval of the study protocol must also be provided in the manuscript.

Field-specific reporting

Please select the one below that is the best fit for your research. If you are not sure, read the appropriate sections before making your selection.

☒ Life sciences ☐ Behavioural & social sciences ☐ Ecological, evolutionary & environmental sciences

For a reference copy of the document with all sections, see nature.com/documents/nr-reporting-summary-flat.pdf

Life sciences study design

All studies must disclose on these points even when the disclosure is negative.

Sample size	No statistical methods were used to determine the sample size. Sample sizes were determined based on optimization experiments, previous in vivo experiments, and published literature which showed that the use of 5 to 10 mice per group was sufficient for the study. Published literature from our lab using the same sample size of mice include (Yang, L. et al. UBXN3B positively regulates STING-mediated antiviral immune responses. Nature Communications 9, 2329, doi:10.1038/s41467-018-04759-8 (2018).PMID: 29899553) and (Yang, D. et al. A critical role for MSR1 in vesicular stomatitis virus infection of the central nervous system. iScience 24, 102678, doi:https://doi.org/10.1016/j.isci.2021.102678 (2021).PMID: 34169243)
Data exclusions	No data was excluded.
Replication	All in vitro experiments were performed at least two-three times and in vivo experiments at least two times as independent experiments and were successfully replicated. This is indicated in the methods and figure legends.
Randomization	No randomization was performed for in vivo mouse studies. Sex- and age-matched littermater mice were assigned to different experimental groups. For in vitro cell work, randomization was not relevant as all cell lines came from the same stock provided by the company and all cells used came from the same passage. This also applies for primary cells isolated from mice eg., MEF cell or bone-marrow derived macrophages, as all cells were generated by the same method and used on the same day of differentiation for experiments. All in vitro cell culture works were preformed under the same conditions for comparison.
Blinding	Researchers were not blinded to experimental conditions and groups during data collection and/or analysis, this is because blinding was not required. All the data presented are based on objective assessments of capture data without subjective assessments.

Reporting for specific materials, systems and methods

We require information from authors about some types of materials, experimental systems and methods used in many studies. Here, indicate whether each material, system or method listed is relevant to your study. If you are not sure if a list item applies to your research, read the appropriate section before selecting a response.

Materials & experimental systems

n/a	Involved in the study	
[ ]	[x]	Antibodies
[ ]	[x]	Eukaryotic cell lines
[x]	[ ]	Palaeontology and archaeology
[ ]	[x]	Animals and other organisms
[x]	[ ]	Clinical data
[x]	[ ]	Dual use research of concern
[x]	[ ]	Plants

Methods

n/a	Involved in the study	
[ ]	[x]	ChIP-seq
[x]	[ ]	Flow cytometry
[x]	[ ]	MRI-based neuroimaging

Antibodies

Antibodies used

GAPDH (Cat# 60004-1-Ig, 1:2000, Proteintech Group, Inc)
GFP (Cat # 50430-2-AP, 1:1000, Proteintech Group, Inc)
UBR5 (Cat# 22782-1-AP, 1:200 for IF, Proteintech Group, Inc)
TRIM28 antibody (20A1, Cat # 619302, 1:1000, BioLegend)
UBR5 antibody (Cat# A300-573A, 1:1000, Bethyl Laboratories, Inc)
Tubulin (Cat# 2144, 1:2000, Cell Signaling Technology)
Actin (Cat#4967, 1:2000, Cell Signaling Technology)
K63-linked polyubiquitin (D7A11, Cat# 5621,1:500, Cell Signaling Technology)
K48-linked polyubiquitin (D9D5, Cat# 12805, 1:1000, Cell Signaling Technology)
SUMO-1 (Cat# 4930, 1:500, Cell Signaling Technology)
SUMO -2/3 (18H8, Cat# 4971, 1:500, Cell Signaling Technology)
His-Tag (D3I1O, Cat# 12698, 1:1000, Cell Signaling Technology)
HA-Tag (C29F4, Cat# 3724, 1:1000, Cell Signaling Technology)
RIG-I (D14G6, Cat# 3743, 1:1000, Cell Signaling Technology)
MDA5 (D74E4, Cat# 5321, 1:1000, Cell Signaling Technology)
MAVS (D5A9E, Cat# 24930, 1:1000, Cell Signaling Technology)
UBR5 (D6O8Z, Cat# 65344, 1:1000, Cell Signaling Technology)
IRF3 (D6I4C, Cat # 11904, 1:1000, Cell Signaling Technology)
STING (D2P2F, Cat#13647, 1:1000, Cell Signaling Technology)
Myc antibody (9E10, Cat# TA150121, 1:1000, OriGene Technologies, Inc)
FLAG (Clone M2, Cat # F1804-200UG, 1:1000, Sigma-Aldrich)
VSV-G antibody (Cat# V4888, 1:1000, Sigma-Aldrich)
Anti-rabbit IgG, HRP-linked Antibody (cat#7074, 1:5000 dilution, Cell Signaling Technology)
Anti-mouse IgG, HRP-linked Antibody (cat#7076 , 1:5000 dilution, Cell Signaling Technology)
Donkey anti-Mouse Alexa Fluor™ Plus 488 (Cat# A32766, 1:200, Thermo Fisher)
Goat anti-rabbit Alexa Fluor™ Plus 594 (Cat# A32740, 1:200, Thermo Fisher)
DAPI Solution (Cat# 62248, 1:1000, Thermo Fisher).

Validation

Validation of the antibodies are available on the manufacturer's website.
GAPDH (https://www.ptglab.com/products/GAPDH-Antibody-60004-1-Ig.htm)
GFP (https://www.ptglab.com/products/eGFP-Antibody-50430-2-AP.htm)
UBR5 (https://www.ptglab.com/products/UBR5-Antibody-22782-1-AP.htm)
TRIM28 antibody (https://www.biolegend.com/en-us/search-results/purified-anti-tif1beta-kap-1-trim28-antibody-1988?GroupID=GROUP26)
UBR5 antibody (https://www.fortislife.com/products/primary-antibodies/rabbit-anti-edd1-antibody/BETHYL-A300-573)
Tubulin (https://www.cellsignal.com/products/primary-antibodies/a-tubulin-antibody/2144)
Actin (https://www.cellsignal.com/products/primary-antibodies/b-actin-antibody/4967)
K63-linked polyubiquitin (https://www.cellsignal.com/products/primary-antibodies/k63-linkage-specific-polyubiquitin-d7a11-rabbit-mab/5621)
K48-linked polyubiquitin (https://www.cellsignal.com/products/antibody-conjugates/k48-linkage-specific-polyubiquitin-d9d5-rabbit-mab-hrp-conjugate/12805)
SUMO-1 (https://www.cellsignal.com/products/primary-antibodies/sumo-1-antibody/4930)
SUMO -2/3 (https://www.cellsignal.com/products/primary-antibodies/sumo-2-3-18h8-rabbit-mab/4971)
His-Tag (https://www.cellsignal.com/products/primary-antibodies/his-tag-d3i1o-xp-rabbit-mab/12698)
HA-Tag (https://www.cellsignal.com/products/primary-antibodies/ha-tag-c29f4-rabbit-mab/3724)
RIG-I (https://www.cellsignal.com/products/primary-antibodies/rig-i-d14g6-rabbit-mab/3743)
MDA5 (https://www.cellsignal.com/products/primary-antibodies/mda-5-d74e4-rabbit-mab/5321)
MAVS (https://www.cellsignal.com/products/primary-antibodies/mavs-d5a9e-rabbit-mab/24930)
UBR5 (https://www.cellsignal.com/products/primary-antibodies/ubr5-d6o8z-rabbit-mab/65344)
IRF3 (https://www.cellsignal.com/products/primary-antibodies/irf-3-d6i4c-xp-rabbit-mab/11904)
STING (https://www.cellsignal.com/products/primary-antibodies/sting-d2p2f-rabbit-mab/13647)
Myc antibody (https://www.origene.com/catalog/antibodies/tag-antibodies/ta150121-1/9e10-anti-myc-monoclonal-antibody)
FLAG (https://www.sigmaaldrich.com/US/en/product/sigma/f1804)

	VSV-G antibody (https://www.sigmaaldrich.com/US/en/product/sigma/v4888)<br>Anti-rabbit IgG, HRP-linked Antibody (https://www.cellsignal.com/products/secondary-antibodies/anti-rabbit-igg-hrp-linked-antibody/7074)<br>Anti-mouse IgG, HRP-linked Antibody (https://www.cellsignal.com/products/secondary-antibodies/anti-mouse-igg-hrp-linked-antibody/7076)<br>Donkey anti-Mouse Alexa Fluor™ Plus 488 (https://www.thermofisher.com/antibody/product/Donkey-anti-Mouse-IgG-H-L-Highly-Cross-Adsorbed-Secondary-Antibody-Polyclonal/A32766)<br>Goat anti-rabbit Alexa Fluor™ Plus 594 (https://www.thermofisher.com/antibody/product/Goat-anti-Rabbit-IgG-H-L-Highly-Cross-Adsorbed-Secondary-Antibody-Polyclonal/A32740)<br>DAPI Solution (https://www.thermofisher.com/order/catalog/product/62248).

Eukaryotic cell lines

Policy information about cell lines and Sex and Gender in Research

Cell line source(s)	2fTGH-ISRE-Luc line was generated previously in Wang lab.<br>A549 (human lung carcinoma epithelial cell, Cat# CCL-185),ATCC<br>Calu-3 (human lung adenocarcinoma epithelial cell, Cat# HTB-55), ATCC<br>HEK293T cells (human embryonic kidney, Cat# CRL-3216), ATCC<br>Vero cells (monkey kidney epithelial cells, Cat# CCL-81), ATCC<br>L929 cells (mouse fibroblast cells, Cat# CCL-1), ATCC<br>Mouse embryonic fibroblasts (MEFs) and Bone marrow derived macrophages (BMDM) were primarily isolated in Wang lab.
Authentication	The cell lines were authenticated from the manufacturer or from the laboratory of origin.The cell lines were frequently checked in the lab by their morphological features.
Mycoplasma contamination	The cells are routinely treated with MycoZAP (Lonza, Basel, Switzerland) and were not tested for Mycoplasma contamination in the lab.
Commonly misidentified lines<br>(See ICLAC register)	No misidentified cell lines were used.

Animals and other research organisms

Policy information about studies involving animals; ARRIVE guidelines recommended for reporting animal research, and Sex and Gender in Research

Laboratory animals	All the animal procedures were approved by the Institutional Animal Care and Use Committee (IACUC) at UConn Health adhering to federal and state laws. . Both male and female mice of age 8 weeks old were used in analyses for all the following strains: Ubr5flox/flox was generated recently by Prof. Robert E. Hill on a background of C57BL/6J and crossed with a tamoxifen inducible ERT2-Cre recombinase line (The Jackson Laboratory, Stock # 008463,background: C57BL/6J), to generate ERT2-Cre+/- Ubr5flox/flox mice. To induce global Ubr5 knockout in >6 weeks old mice, 1 mg of tamoxifen (dissolved in corn oil) was administered to each animal every other day, totaling five times. ERT2-Cre+/- Ubr5flox/flox mice treated with corn oil served as the wild-type control (Ubr5+/+). Tamoxifen was cleared for two weeks after the last dose. These mouse strains were housed in the specific pathogen-free animal facility at UConn Health. All mice were housed at an ambient temperature of approximately 24 °C, a humidity of 40~60%, and a light/dark cycle of 12 hours. Details of mice strains used and all animal procedures are described in the methods sections of the manuscript.
Wild animals	No wild animals were used in the study.
Reporting on sex	Both male and female mice were included in the study and the findings apply to both sexes.
Field-collected samples	Did not involve samples collected from the field.
Ethics oversight	The animal studies were approved by the UConn Health Institutional Animal Care and Use Committee (IACUC).

Note that full information on the approval of the study protocol must also be provided in the manuscript.

Plants

Seed stocks	N/A
Novel plant genotypes	N/A
Authentication	N/A

ChIP-seq

Data deposition

☒ Confirm that both raw and final processed data have been deposited in a public database such as GEO.

☒ Confirm that you have deposited or provided access to graph files (e.g. BED files) for the called peaks.

Data access links<br>*May remain private before publication.*	To review GEO accession GSE245604:<br>Go to https://www.ncbi.nlm.nih.gov/geo/query/acc.cgi?acc=GSE245604
Files in database submission	GSE245602,GSE245603
Genome browser session<br>(e.g. UCSC)	https://www.ncbi.nlm.nih.gov/geo/query/acc.cgi?acc=GSE245604

Methodology

Replicates	duplicate mixed.
Sequencing depth	Libraries were sequenced on an NovaSeq sequencing and 1 2x150bp reads were generated
Antibodies	Mouse anti-TRIM28 antibody (8 µg/reaction, BioLegend, Cat # 619302)
Peak calling parameters	Peaks were identified by comparing the ChIP and input data using MACS2 v2.1.2. The peaks were annotated by their proximity to transcription start site of the corresponding genes, ranging from -3 to +3kb in the sample.
Data quality	Paired-end NovaSeq sequencing was analyzed based on the barcoded libraries following the manufacturer's description. ChIP-seq reads were aligned to human reference assembly GRCh38 using Bowtie2 v2.3.5.1 aligner. 2x150bp, ~150M PE reads, 25M reads per sample.
Software	Bowtie2 v2.3.5.1 aligner, MACS2 v2.1.2
